# Personalized dose selection for the first Waldenström macroglobulinemia patient on the PRECISE CURATE.AI trial

**DOI:** 10.1038/s41746-024-01195-5

**Published:** 2024-08-27

**Authors:** Agata Blasiak, Lester W. J. Tan, Li Ming Chong, Xavier Tadeo, Anh T. L. Truong, Kirthika Senthil Kumar, Yoann Sapanel, Michelle Poon, Raghav Sundar, Sanjay de Mel, Dean Ho

**Affiliations:** 1 The Institute for Digital Medicine (WisDM), National University of Singapore, Singapore, 117456 Singapore; 2 The N.1 Institute for Health (N.1), National University of Singapore, Singapore, 117456 Singapore; 3 Department of Biomedical Engineering, College of Design and Engineering, National University of Singapore, Singapore, 117583 Singapore; 4 Department of Pharmacology, Yong Loo Lin School of Medicine, National University of Singapore, Singapore, 117600 Singapore; 5 Department of Medicine, Yong Loo Lin School of Medicine, National University of Singapore, Singapore, 119228 Singapore; 6 Department of Haematology-Oncology, National University Cancer Institute (NCIS), National University Hospital, Singapore, 119228 Singapore; 7 Singapore Gastric Cancer Consortium, Department of Medicine, National University of Singapore, Singapore, 119228 Singapore; 8 The Bia-Echo Asia Centre for Reproductive Longevity and Equality (ACRLE), National University of Singapore, Singapore, 117456 Singapore; 9 Present Address: Roche Information Solutions, Santa Clara, California, USA

**Keywords:** Haematological cancer, Translational research

## Abstract

The digital revolution in healthcare, amplified by the COVID-19 pandemic and artificial intelligence (AI) advances, has led to a surge in the development of digital technologies. However, integrating digital health solutions, especially AI-based ones, in rare diseases like Waldenström macroglobulinemia (WM) remains challenging due to limited data, among other factors. CURATE.AI, a clinical decision support system, offers an alternative to big data approaches by calibrating individual treatment profiles based on that individual’s data alone. We present a case study from the PRECISE CURATE.AI trial with a WM patient, where, over two years, CURATE.AI provided dynamic Ibrutinib dose recommendations to clinicians (users) aimed at achieving optimal IgM levels. An 80-year-old male with newly diagnosed WM requiring treatment due to anemia was recruited to the trial for CURATE.AI-based dosing of the Bruton tyrosine kinase inhibitor Ibrutinib. The primary and secondary outcome measures were focused on scientific and logistical feasibility. Preliminary results underscore the platform’s potential in enhancing user and patient engagement, in addition to clinical efficacy. Based on a two-year-long patient enrollment into the CURATE.AI-augmented treatment, this study showcases how AI-enabled tools can support the management of rare diseases, emphasizing the integration of AI to enhance personalized therapy.

## Introduction

Digitalization is increasingly influencing healthcare systems as it holds promise to improve healthcare accessibility, scalability, and data flow^1^. Patients are increasingly accepting digital health: the COVID-19 pandemic accelerated the adoption of telemedicine, and the use of wearables for fitness and health monitoring is becoming more common^2^. Despite the challenges involved, physicians are embracing digital possibilities^3^. With digitized health data, artificial intelligence (AI) becomes a fundamental part of the analysis, discovery, and personalization of medical care^4^; clinical decision support systems (CDSS), smart devices, and sensors singularly have the potential to benefit from the AI application^5^.

In oncology, AI was also recognized to have great potential, and evolutionary game theory has been proposed to personalize cancer therapy in a dynamic fashion, by anticipating the future characteristics of the tumor^6,7^. While AI, especially machine learning, is promising, challenges remain for its use in rare and orphan diseases. Among those obstacles are a shortage of extensive, well-annotated datasets and a restricted patient population^5^. An alternative to the big data approach can be highly advantageous.

One example of small data CDSS is CURATE.AI^8^, an indication agnostic, deterministic, AI-derived platform being validated in cancer^9^, immunosuppression^10^, and even cognitive training^11^. The quadratic correlations between drug doses and biological responses were first discovered through neural network models^9^. Three-drug combinations across eight unique doses and the measured biological responses in in vitro models were used to train, test, and validate the mathematical models. Developed based on the neural network-derived observations^9^, further robust optimization and validation studies were performed in vitro^12,13^, in vivo^14,15^, ex vivo^16,17^, and in retrospective patient data^18–20^ over the years. Recently, prospective/interventional clinical trials for other indications and different workflows have been conducted for feasibility (NCT03759093, NCT04357691, NCT05376683, NCT04848935, and NCT05175235)^21^. Currently based on a small set of patient data, CURATE.AI operates by dynamically calibrating an N-of-1 profile that identifies the optimal treatment intensity (e.g., drug dose, training intensity) for a desired phenotypic output (e.g., tumor reduction, cognitive improvement). This objective aligns with Project Optimus, an FDA-led plan to reform dose optimization criteria in oncology. The project advocates for dose determination using both nonclinical and clinical data to minimize harm and maximize advantages for patients (https://www.fda.gov/about-fda/oncology-center-excellence/project-optimus).

Waldenström macroglobulinemia (WM) is a rare mature B-cell malignancy characterized by clonal lymphoplasmacytic (LPL) bone marrow infiltration and monoclonal immunoglobulin M (IgM)^22^. While a “watch and wait“ approach is recommended for asymptomatic patients, therapy is indicated when the IgM paraprotein or LPL infiltrate results in clinical manifestations^23^. The key classes of drugs commonly used for the treatment of WM include monoclonal antibodies, alkylating agents, proteasome inhibitors, and Bruton Tyrosine Kinase inhibitors (BTKi)^24^. Rituximab-based immunotherapy and BTKi are currently the favored frontline treatment options for WM^25^.

Given the advanced age of the typical WM patient, treatment-related toxicities are an important consideration. There are currently no validated CDSS platforms for individualized dosing of BTKi in hematologic malignancies. Dose adjustments are made subjectively based on physicians' discretion, usually after the occurrence of toxicities. The cost-effectiveness of current dosing strategies for BTKi is also questioned, given that these drugs are typically used long-term. In the USA, Ibrutinib (Imbruvica) is one of the ten drugs selected by Medicare for initial price negotiation (https://www.cms.gov/inflation-reduction-act-and-medicare/medicare-drug-price-negotiation), which illustrates its health economic significance.

CDSS for individualized drug dosing in WM would be a valuable clinical tool. WM has an incidence rate of approximately three out of every 1,000,000 people per year. Similar to the case of other very rare diseases, it creates a considerable challenge and cost to recruit a large patient population for a clinical trial^26^^,27^. In addition, the existence of high heterogeneity among individuals within the small population further increases treatment challenges. In this case report, CURATE.AI addresses the challenge of high patient heterogeneity^26–29^ by only using the patient’s own data to guide their own treatment. Unlike existing big data approaches, no model training and testing with population data are required for small data approaches such as CURATE.AI. This case report describes the interim results from two years of treatment of the first patient enrolled under the WM cohort of the PRECISE CURATE.AI clinical trial. CURATE.AI was intended as CDSS supporting physicians in their decision-making. CURATE.AI dynamically generated Ibrutinib dose recommendations that aimed to optimize response based on serum IgM levels, considering patient toxicities. We performed a preliminary evaluation of the feasibility of this setup through measures of patient engagement, dose adherence, cumulative dose per cycle against standard-of-care and adverse events as well as preliminary discussion on the impact dimensions.

## Results

### Patient characteristics and diagnostic assessment

The patient was an 80-year-old male with an ECOG performance status of 1 and no significant comorbidities. He was diagnosed with WM in 2019 based on IgM paraproteinemia confirmed on serum immunofixation, and a bone marrow aspirate and biopsy confirming a clonal LPL infiltrate. The MYD88 L265P mutation was detected by PCR on the bone marrow sample. His computed tomography scan showed no lymphadenopathy, and his renal and liver functions were normal. He was initially asymptomatic with a mild anemia of 9–10 g/dL. He developed worsening anemia in the subsequent months requiring initiation of treatment. After discussing potential treatment options, the patient provided consent and was enrolled in the trial in October 2021. His IgM pre-treatment was 48.3 g/L and his hemoglobin had dropped to 6.5 g/dL.

### CURATE.AI—supported Ibrutinib treatment

**Fig. 1:**
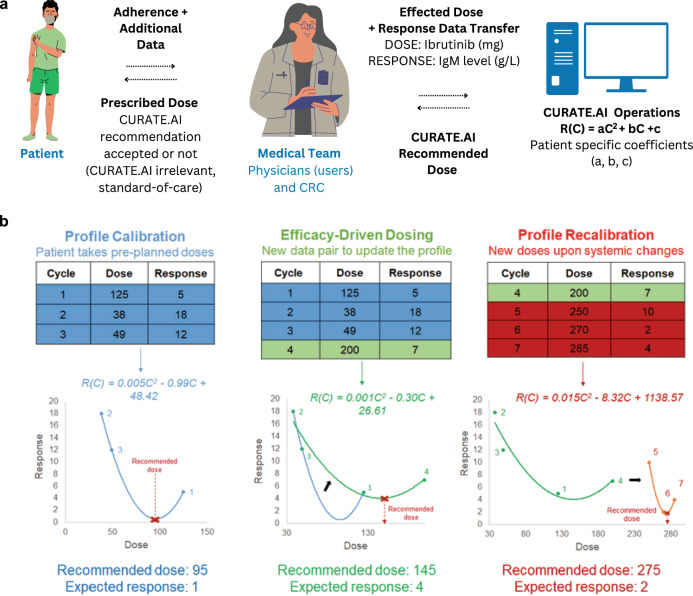
CURATE.AI operations. **a** CURATE.AI is designed as a clinical decision support system, providing recommendations to physicians who are the users of CURATE.AI. New data from the patients (adherence) and from the healthcare system (patient results) are used to dynamically update patient profiles. **b** Phases of CURATE.AI include calibration and efficacy-driven dosing. Additionally, if systemic changes in the patient dose-response profile are expected/detected the profile can go through a recalibration. CRC clinical research coordinator.

**Fig. 2:**
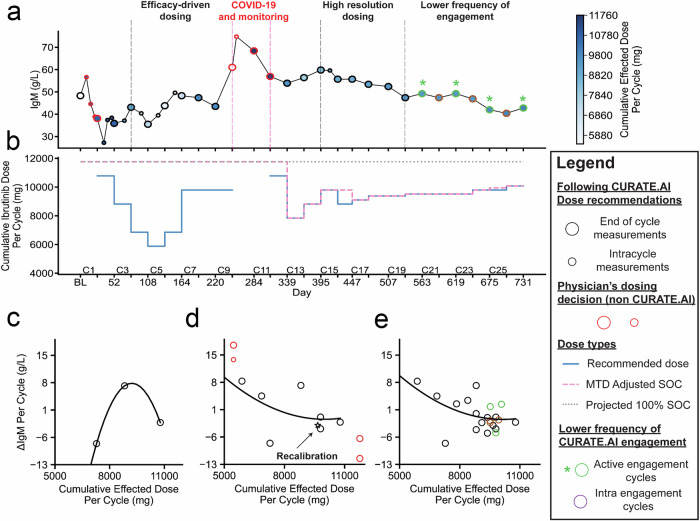
CURATE.AI-assisted Ibrutinib dose decision-making according to the resulting IgM level over two years of the patient enrollment. **a** Patient IgM levels and the corresponding, effected Ibrutinib doses (represented as hues of blue). **b** Comparison between CURATE.AI-recommended doses and standard-of-care doses. **c**–**e** Evolving patient dose-response profiles, from the initial calibration, to post-Covid-19 recalibration and final patient profile after two years of enrollment. C* cycle number, MTD maximum tolerated dose, SOC standard-of-care, BL baseline level.

In the profile calibration phase, the CURATE.AI team proposed to the physicians drug doses across the prespecified safety range. Based on the patient’s [dose:response] data pairs, a CURATE.AI profile was generated. During the efficacy-driven dosing phase, physicians were provided with recommendations of optimal doses based on the patient’s profile and within the prespecified safety dose range. The corresponding patient’s response was paired with the effected dose and was used to update the profile. When the patient experienced a systemic change, the profile was recalibrated with new data pair (Fig. 1). The physicians themselves were deeply involved in prospectively building the small dataset that was then used for the optimal dose recommendation. Three physicians (users) participated in the patient’s treatment decisions (Fig. 1). Ibrutinib was administered once a day with doses ranging between 420, 280, and 140 mg, which were adjusted weekly (Supplementary Fig. 1). The dose in cycle one was decided by the physicians as they only considered one dose to be appropriate as the initial dose (e.g., the safety range included only one dose). CURATE.AI engagement for that dose selection decision was considered irrelevant. CURATE.AI provided dose recommendations to the physicians for the next two cycles with the calibration intent. The first three cycles constituted the calibration phase (Fig. 1a). The patient’s IgM demonstrated significant intracycle variability (Fig. 2a). Within this phase, Ibrutinib doses oscillated between 61.9–91.7% (7280–10,780 mg/cycle) of the established standard-of-care (100% = 11760 mg/cycle) (Fig. 2b). The shape of the profile evolved with the new data pairs incorporated at each subsequent cycle (Fig. 2c–e).

During cycle nine, Ibrutinib treatment was paused due to COVID-19 infection. The dose selection for the next two cycles was done by the users without the assistance of CURATE.AI and the cycle 9–11 data pairs were excluded from the profile to limit the potential noise from the response data collected during the systemic change. CURATE.AI dose recommendation for cycle 12 had a recalibration intent. The resulting ΔIgM had a good alignment with the profile (1.59 g/L prediction error) in the dose range of interest, and instead of proceeding with the full recalibration, the efficacy-driven dosing restarted (Fig. 2d).

The patient experienced dose-limiting toxicities in cycle 12 (Grade 2 Epistaxis) and the dose range available for CURATE.AI recommendations was limited. Encouraged by high patient adherence, the dose adjustment changed from weekly to daily starting from cycle 15 (Supplementary Fig. 1); CURATE.AI was adapted to provide recommendations with increased dosing resolution. From cycle 20 onwards, the CURATE.AI dose recommendation was provided for two consecutive cycles as the patient’s condition was considered stable. During the dose selection for cycle 25, the users overrode the CURATE.AI recommendation made from cycle 24 in accordance with an increased maximum tolerated dose (MTD) in cycle 25. MTD is defined as the highest dose that an individual patient can tolerate, given the evoked toxicity. Based on the patient’s history and past cycles, the physicians determine the MTD and decide on the daily titrated doses within the cycle. A follow-up appointment is conducted at the end of each cycle to assess the patient’s toxicities and determine the MTD for the next cycle. As such, the MTD for Ibrutinib in this study was determined through the physicians' assessment of the likelihood of the dose-dependent toxicities.

Over two years, CURATE.AI’s accuracy improved, moving from a median 2.7 (IQR 1.7–14.0) g/L absolute prediction error in cycles 4–8, to 2.2 (IQR 1.4–3.6) g/L in cycles 12 to 20 and 2.2 (IQR 0.5–3.4) g/L in cycles 21–26. Of note, starting at cycle 14, the CURATE.AI dose recommendation predominantly overlapped with MTD-adjusted standard-of-care. The flat, horizontal shape of the profile at the high doses indicated that at that dose range, the increase in the dose led only to a limited ΔIgM change.

Using CURATE.AI added two steps to the clinical workflow: involving the CURATE.AI team and informing the patient (via verbal communication and a written dosing calendar) of the dose adjustment before the next cycle. CURATE.AI was considered relevant in 88.5% (23/26) cycles and the CURATE.AI recommendations were accepted by the users for prescription in 95.7% (22/23) of those instances, demonstrating high human-computer agreement. In four cycles (9, 12, 15 and 25), the users adjusted Ibrutinib dose intracycle (Supplementary Fig. 1). Cumulatively, over the two years of patient enrollment, CURATE.AI-recommended dose was different by 22.3% (60,200 mg) from the projected standard-of-care, and by 11.6% (27,580 mg) from the MTD-adjusted standard-of-care. Additional assessments of CURATE.AI are included in the Supplementary Results.

### Clinical outcomes

**Fig. 3:**
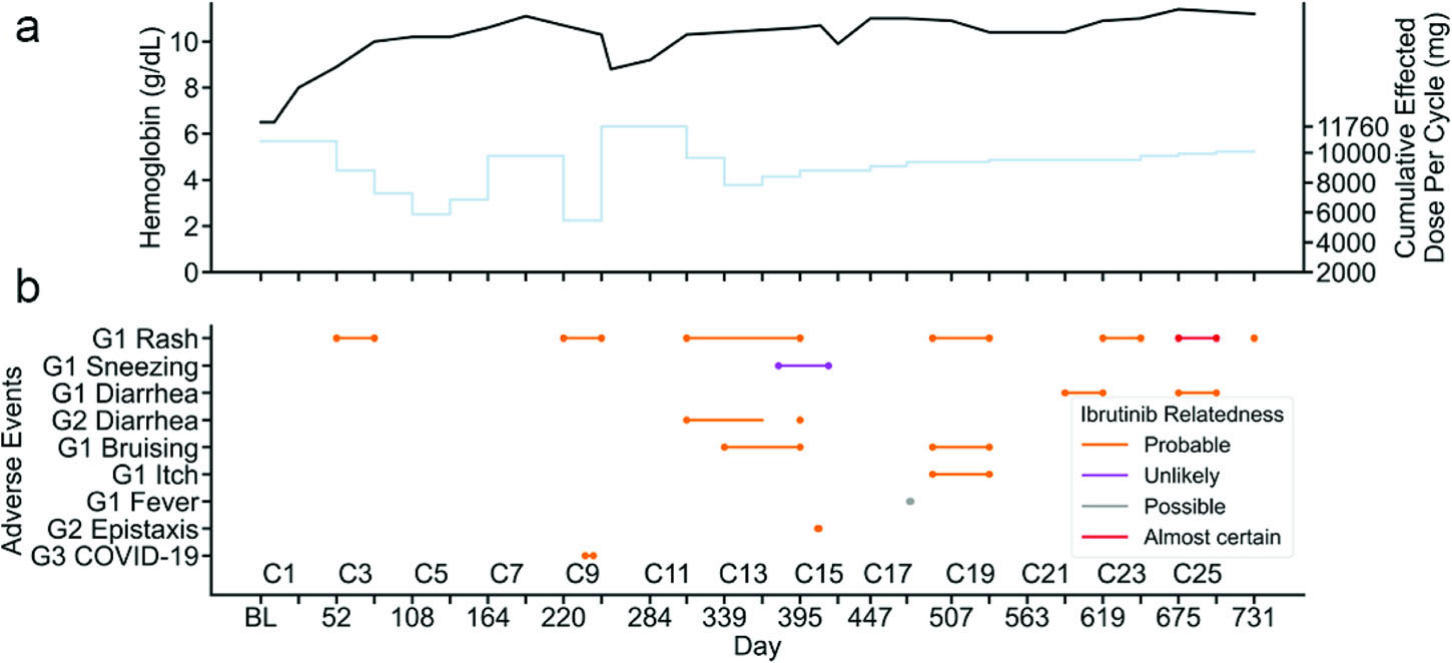
Clinical outcomes of CURATE.AI-assisted Ibrutinib dose decision-making over two years of the patient enrollment. **a** Patient hemoglobin levels (black) and the corresponding effected Ibrutinib doses (blue). **b** Adverse events throughout the course of the patient engagement as reported by the patient at the time of their clinical visits. C* cycle number, BL baseline level.

The patient showed a 99.2% (722/728 days) adherence as measured by basic pharmacovigilance at the regular clinical visits. The non-adherence occurred in cycle four, when the patient delayed switching from the high dose (420 mg) to the medium dose (280 mg) by three days, and in cycle 14 when the patient adjusted the dose himself for three days due to the experienced side effects (Supplementary Fig. 1). The treatment was well tolerated. Over the two-year-long trial engagement, the patient experienced rare grade two toxicities (epistaxis and diarrhea) and grade one toxicities (rash, which resolved with topical therapy, self-limiting diarrhea, bruising, and itch) with probable relatedness to Ibrutinib (Fig. 3b). Additionally, the patient contracted COVID-19, and it is probable that Ibrutinib made them more susceptible to the infection. From cycle 15, the daily dose variations between high (420 mg) and medium (280 mg) doses might have affected side effects compared to the previous weekly adjustments (Supplementary Fig. 1).

The patient’s IgM level demonstrated an 11.4% (5.5 g/L) reduction from baseline to the level measured at the two-year mark. The response at the two-year mark fell short of the minor response criteria and qualified for stable disease (SD) based on IWWM criteria^30^. The maximal IgM reduction from the baseline was 43.6% (21.1 g/L) noted after week one of cycle two, which increased back to the near pre-cycle level within a week (Fig. 2a). A rapid rise in the IgM level was noted during the pause of treatment at the time of the COVID-19 infection (Fig. 2a). The hemoglobin level rose to 10 g/dL by cycle three and was maintained between 10–11 g/dl throughout the engagement except for the dip in during the COVID-19-related treatment pause (Fig. 3a).

The patient did not require hospital admission throughout the two-year engagement except for during his COVID-19 infection. Although no formal patient-reported outcomes were collected, the patient remained fully independent in his activities of daily living and reported a willingness to continue with the trial engagement. Similar sentiments were shared by the patient’s caregiver.

## Discussion

We present the first application of CDSS for individualized dosing of BTKi in WM. Importantly, we demonstrated the feasibility of performing the necessary response assessments and cycle-by-cycle dose adjustments based on CURATE.AI in an elderly patient. It is noteworthy that the patient did not achieve a deep response based on a reduction in IgM. However, the significant improvement in hemoglobin, and good quality of life enjoyed by the patient over the two years is arguably more important. Indeed, WM is an incurable malignancy and the depth of response by IWWM criteria may not necessarily translate into a progression free survival benefit^31^. Prioritizing symptom control, quality of life, and minimizing toxicity is, hence, a reasonable strategy, especially in the elderly, who comprise the majority of patients with WM.

The rapid rise in IgM associated with the COVID-19 infection may have been related to the treatment interruption. However, COVID-19 is known to have a plethora of immunomodulatory effects^32^ and it is possible that these may have played a part in the IgM rise. With COVID-19 becoming an endemic infection worldwide, its impact on the tumor microenvironment and immune surveillance of WM is an important area to be addressed by future research.

Evidence is growing about the variability in interpatient pharmacokinetics and the limitations of uniformly treating patients with anticancer drugs at standard-of-care/label doses^33^. Small data solutions for personalized care have advantages over big or complex data approaches and can help move beyond “pilotitis”^34^. In the context of this trial, and this case report, the small dataset behind CURATE.AI operations not only enabled personalized treatment of the patient with a rare disease but also facilitated dynamic treatment adaptation to his unique circumstances and systemic changes such as elevated IgM levels post-COVID-19. Small data aligns with patients’ desire for personalization, while avoiding extensive data collection and issues like algorithmic bias. It can also enhance interoperability and integration into diverse healthcare systems, influencing digital health uptake^1,35^. Finally, the discussion in healthcare innovation and AI in medicine increasingly involves the required computational power and related carbon emissions^36^. Reflecting a move towards greener IT solutions, CURATE.AI transitioned from a more computationally expensive neural network computation to a computation-light quadratic [dose:response] approach, in line with trends like tiny machine learning and compact AI^1^.

Stakeholder inclusion and patient-centered care are emerging as best practices in digital health innovation and implementation^1^. The co-creation with physicians was pointed out as foundational in bridging AI fairness and meaningful clinical benefits^37^. Additionally, user inclusion can ameliorate the uncertainty around the reliability of the underlying data—one of the commonly voiced concerns^38^. PRECISE CURATE.AI involved users in both the study design, with physicians as part of the team^39^, and the co-creation of the patient profile dataset. Accordingly, in an additional behavioral study on physicians' sentiments toward CURATE.AI, we identified collaborative functioning and data availability as some of the key aspects that define their technology adoption^40^. Another key aspect was patient safety. In this regard, the CURATE.AI workflow positions the users as the ultimate decision-makers. Over two years, users found CURATE.AI irrelevant in 3/26 cycles and rejected its recommendation in 1/26 cycles, showing strong utilization and agreement. The users were also free to amend the dose intracycle as per standard-of-care and did so for 4/26 cycles (Supplementary Fig. 1).

While we have not included patients in the co-creation of the study or formally sought their feedback, his adherence may serve as a proxy in gauging his sentiments^41^. 99.2% (722/728 days) adherence to varying weekly and daily doses of Ibrutinib is indicative that the patient had a high perceived value of the treatment. Over the two years of engagement, he had an opportunity to learn about the treatment value through experiencing the treatment and its trade-offs (effectiveness vs. side effects and cost) and chose to adhere to it over non-adherence or switching to alternative treatments. Finally, new models are being proposed to reconcile patient voice and physician expert judgment with the robustness of AI-based decision-making. In one example, Cohen et al. drew from judiciary processes to propose human involvement and AI decision review only on appeal^42^. The utility and implementation of such models in healthcare is yet to be demonstrated.

CDSSs are considered among the top three investment priorities in digital health, together with digital connectivity infrastructure and telemedicine (https://transformhealthcoalition.org/wp-content/uploads/2022/10/Closing-the-digital-divide-mainReport.pdf). When evaluating the impact of CDSS from both health and economic perspectives, it is crucial to recognize them as complex interventions. As a result, their potential impact on clinical, process, and patient-reported outcomes may be complex^43^. For example, not only primary outcomes directly influenced by CDSS-driven decisions should be considered but also secondary outcomes, which may be affected, for instance, by the reallocation of healthcare resources^43^.

According to Wright et al.‘s clinical decision support taxonomy^44^, CURATE.AI falls into the front-end clinical decision support intervention category for medication dosing support. The benefits of such a system are manifold, ranging from improved patient outcomes and error prevention to enhanced decision support and process improvement^45^. CURATE.AI represents an incremental innovation in personalized medicine, and while it is early in its innovation lifecycle, some of the evidence of its impact can be already gleaned.

CURATE.AI offers a structure to a passive personalized medicine, where patient response characteristics are learned exposing the patient to a range of doses^46^. This structure streamlines the process of the treatment as an experience good (i.e., a service that is experienced as opposed to owned) and lowers the related costs (understood broadly) by limiting the duration of the learning process (i.e., calibration phase). Additionally, it enables dynamic adjustment of the dose throughout the treatment duration.

From a health impact perspective, Hult (2014) highlighted that changes in dosages, the primary outcome of CURATE.AI, account 60% of FDA-approved incremental innovations^47^. The current 100% standard-of-care Ibrutinib dose is 420 mg daily, which accounts for the cumulative dose of 11,760 mg in a 28-day cycle. In this study, the cumulative dose in a 28-day cycle recommended by CURATE.AI was either the same or lower than the MTD-adjusted standard-of-care (Fig. 2b). The total dose difference between the CURATE.AI and MTD-adjusted standard-of-care approach was 11.6%. Such changes in dosages could potentially reduce dose-dependent toxicities. The reduction in the toxicities may subsequently encourage patient adherence to treatment^5,6^. In our study, the patient demonstrated 99.2% adherence. Additionally, despite the reduced cumulative doses, we noted stabilization of the patient’s IgM level (Fig. 2a), and a marked improvement in hemoglobin level (Fig. 3a) was achieved.

A comparison of the toxicities experienced by the patient in our study with published toxicity data for Ibrutinib suggests a promising safety profile of CURATE.AI-based Ibrutinib dosing. Notably, the patient did not experience any cardiac toxicity (which has been reported in over 10% of patients treated with Ibrutinib in clinical trials^48^). Furthermore, he reported no significant skin (reported in 16% of patients) or gastrointestinal toxicities (reported in 6% of patients)^49^. Although a formal comparison of safety with a standard-of-care group should be conducted in the context of a randomized trial, the data emerging from this case report support such investigation.

The information on the expected response to a given dose can be beneficial to both physicians and patients. Patients have become increasingly interested and empowered in the decision-making on their own treatment^50^, and even healthcare design^51^. Daugherty et al. demonstrated that in phase one clinical trials, 76% of oncology patients, given the choice, opted to select the dose of their anticancer drugs^52^. CURATE.AI can provide the basis to further involve patients in the discussions on which dose to choose, given the cost of the IgM improvements in terms of side effects and financial toxicity.

From an economic impact perspective, factors such as healthcare resource utilization, and cost of the pharmaceutical treatment are often reported as having a significant impact on the cost-effectiveness of digital health solutions^53^. In this study, the use of CURATE.AI during the calibration stage resulted in an additional ten blood tests, totaling USD 217. Subsequent to the calibration stage, there was no need for additional consultations, hospital visits, laboratory tests, or scans compared to the standard-of-care. Any potential efficiency gains through CURATE.AI, such as reduced physician time spent on decision-making, would likely be marginal.

The cost of pharmaceutical treatment is another important aspect of the potential economic impact of CDSS for medication dosing support, for three main reasons: (1) drug expenditures account for an increasing proportion of health costs, totaling USD 1.1 trillion in annual expenditure worldwide^54^, (2) drug spending is heavily driven by a relatively small number of high-cost products (https://aspe.hhs.gov/sites/default/files/documents/88c547c976e915fc31fe2c6903ac0bc9/sdp-trends-prescription-drug-spending.pdf), and (3) hundreds of billions of dollars are spent each year on “overtreatment with prescribed medications that are either unnecessary or are in excess of lowest cost-effective therapy”^54^. This explains recent price negotiations for a list of 10 prescription medicines, including Ibrutinib (Imbruvica), by the US Medicare health program (https://www.cms.gov/inflation-reduction-act-and-medicare/medicare-drug-price-negotiation).

In our patient’s case, there was an 11.6% total dose difference between the CURATE.AI and MTD-adjusted standard-of-care approaches, resulting in cost savings of USD 7811 based on retail drug costs over a period of two years for the patient. This has the potential to significantly reduce out-of-pocket expenses for patients in specific countries. While the primary goal of the CURATE.AI approach is not to control pharmaceutical costs, it aims to optimize treatment by considering individual treatment effects and burdens, which can lead to direct cost savings.

From both an economic and clinical standpoint, in a future comprehensive health technology assessment of CURATE.AI, it will be crucial to quantify the healthcare resources required to support its implementation and potential efficiency gains while also considering potential medication errors, complications, and drug-related adverse events.

This case study serves as a starting point for a discussion on personalized medicine in the age of AI. CURATE.AI and this study is not without limitations. Firstly, with the emergence of more sensitive techniques for the measurement of monoclonal proteins (such as mass spectrometry), we look forward to their application in monitoring WM and as readouts for CURATE.AI. Additionally, a single case study is not sufficient to conclude about the potential or feasibility of this technology and the observations made in the treatment of this patient have limited generalizability.

AI holds great promise for improving healthcare quality and access. In this case study, we describe the results of a two-year-long enrollment of the first patient with Waldenström macroglobulinemia into PRECISE CURATE.AI trial—a single-center, single-arm, open-label, pilot feasibility trial. We demonstrate high WM patient and physician adherence to CURATE.AI recommendations, and CURATE.AI’s adaptability to patient circumstances. While CURATE.AI recommendations in the efficacy stage overlapped significantly with MTD-adjusted doses, the CURATE.AI profile revealed the [dose:response] dynamics that supported informed decision-making by the physicians and had the potential to enhance patient involvement in balancing the IgM outcomes with the side effects and cost of the treatment.

## Methods

### Research governance and reporting standards

The patient gave informed consent and was recruited under the PRECISE CURATE.AI trial (registration no. NCT04522284 at clinicaltrials.gov) approved under NHG DSRB 2020/00334 and NUS IRB 2021-671. All procedures followed the Helsinki Declaration of 1975, as revised in 2008. The study is reported according to DECIDE-AI guidelines^55^ and additionally includes reporting elements from CARE guidelines^56^. The users of CURATE.AI were physicians who treated the patient and joined the trial as co-investigators. Users, but not patients, participated in the development and research-relevant conduct of the study.

### Study design

PRECISE CURATE.AI, WM Cohort is a single-center, single-arm, open-label, pilot feasibility trial in patients diagnosed with WM and intended for treatment with BTKi, specifically Ibrutinib or Acalabrutinib. CURATE.AI provided Ibrutinib dose recommendations to the users for each four-week cycle operating within the prespecified safety range of 50–100% standard-of-care dose calculated as a total dose per cycle (i.e., not more than 28 days × 420 mg once daily). The users were free to adjust the safety range for each dosing event and to accept or reject the CURATE.AI recommendations. The primary and secondary outcome measures were focused on scientific and logistical feasibility and are presented in Supplementary Table 1; however, this single case report is not intended to categorically assess any of them. Patient’s safety was monitored as per the institutional guidelines—please see the study protocol for details (Supplementary File 1). Recruitment began at Singapore’s National University Hospital at the start of 2021. The study protocol (Supplementary File 1), and the summary of changes since the patient recruitment with reasons (Supplementary Table 2), can be found in the Supplementary Information.

### Clinical decision support system

CURATE.AI algorithmic process used a quadratic relationship correlating dose modulations with the patient’s biomarker responses. The profile generated in the calibration stage was then used to identify the dose that would have provided an optimal biomarker response in the following cycle, towards either reaching maximal biomarker reduction or sustaining its current level, as decided by the users (physicians). The subsequent cycle’s data pair [Ibrutinib dose:ΔIgM], representing the patient’s [dose:response], was added cumulatively to the profile, such that the patient’s profile was continuedly updated and remained relevant to the patient’s state at each time point, throughout their enrollment. If the patient was undergoing systemic changes, either due to the course of their disease and treatment (e.g., biomarker surge, relapse) or due to unrelated reasons (e.g., infectious disease, flare-up of comorbidities), CURATE.AI would undergo recalibration, i.e. collection of new data pair(s) with the intention of a profile recalibration to adjust/redefine the [Ibrutinib dose:ΔIgM] profile (Fig. 1b). This is in contrast to the objective of achieving an optimal efficacy, which is typical for the efficacy-driven dosing phase. Of note, if a systemic change was known to have a temporal character and to be irrelevant to the [dose:response] relationship after the period of a systemic change, the data from the systemic change period were excluded and not used for the profile adjustment.

Supplementary Table 3 lists the data given to the CURATE.AI team. Dose recommendations were provided to the users via a recommendation sheet attached to an email (Supplementary File 2) by the CURATE.AI team. Both users and the CURATE.AI team could request extra data or explanations. Users’ sentiments towards CURATE.AI was evaluated in a separate study via qualitative interviews^40^.

### Treatments and procedures

Key recruitment criteria included age >21, ECOG performance status of 0–2 and a WM diagnosis^57^. Immunoglobulin M paraproteinemia was confirmed by serum immunofixation and the total IgM needed to be at least above two times the upper limit of normal. After the recruitment, patient received Ibrutinib doses selected with the support of CURATE.AI, and underwent longitudinal monitoring and other planned or emergency treatment as per standard-of-care. The research-specific treatments were limited to performing blood draws to quantify IgM and serum-free light chain (sFLC) at a higher frequency for the first 6 cycles. For a full description of recruitment criteria, treatment, and procedures, please see the attached protocol (Supplementary File 1).

### Analytical methods

Datasets with less than 30 data points were considered to have non-normal distribution and are presented as median and interquartile range (IQR).

### Supplementary information

Supplementary Information

Supplementary Data 1

## Data Availability

All data were included in the main manuscript and in the supplementary information. The data-sharing plan at clinicaltrials.gov has changed after the trial registration to include the sharing of anonymized, individual patient data.

## Code availability

Data processing and CURATE.AI analyses were conducted using built-in functions in Python programming language. The custom code can be shared on a reasonable request to Prof. Dean Ho.
