# Supplementary material for: Personalized dose selection for the first Waldenström macroglobulinemia patient on the PRECISE CURATE.AI trial
Source: NPJ Digit Med. 2024 Aug 27;7:223. doi: 10.1038/s41746-024-01195-5 (PMC11350179; doi:10.1038/s41746-024-01195-5)
Supplement: Supplementary file 1 — Supplementary Information [file 41746_2024_1195_MOESM1_ESM.pdf]

Personalized dose selection for the first Waldenström Macroglobulinemia patient on the PRECISE CURATE.AI trial

Agata Blasiak*, Lester W. J. Tan, Li Ming Chong, Xavier Tadeo, Anh T. L. Truong, Kirthika S. Kumar, Yoann Sapanel, Michelle Poon, Raghav Sundar, Sanjay De Mel* , Dean Ho*

Supplementary Information

List of Content:

- **Supplementary Results**
- **Supplementary Figure 1 |** Effected Ibrutinib dosing schedule in each cycle based on pharmacovigilance information.
- **Supplementary Table 1 |** Outcome measures for the PRECISE CURATE.AI pilot study, WM Cohort.
- **Supplementary Table 2 |** Changes made to the protocol after the recruitment of the first patient in PRECISE CURATE.AI pilot study relevant to the WM Cohort.
- **Supplementary Table 3 |** Patient data types used for CURATE.AI operations.
- **Supplementary File 1 |** PRECISE CURATE.AI Trial Protocol, WM Cohort.
- **Supplementary File 2 |** CURATE.AI Recommendation Sheet.
- **Supplementary Data 1 |** Trial data

Supplementary Results

Case Report Results in the Context of the Trial Objectives

CURATE.AI PRECISE trial was intended to assess feasibility of setting up a randomized clinical trial with CURATE.AI. We use the trial outcomes framework to structure the reporting on CURATE.AI in this case report (Table S1). The section below includes the discussion on the pre-specified trial outcomes, unless covered in the main manuscript text.

Primary outcome measures focus on CURATE.AI applicability. The absolute prediction error of CURATE.AI throughout the efficacy-driven phase was 2.19 (IQR 0.84-3.55) g/L and constituting 4.51 (IQR 1.69-7.43) % of the patient's IgM levels. The initial profile and its dynamic iterations were actionable, that is, allowed for dose identification that fulfilled the co-investigators pre-specified safety requirements. Additionally, the dose-response relationship was observed, and the profile was generated within the first 4 cycles, allowing to transition into efficacy-driven dosing early and with more than 20 cycles of potential benefit to the patient by the time of the data cut off at 2 years. Finally, only one systemic change took place for this patient (COVID-19, cycles 9-11) leading to CURATE.AI considered irrelevant for cycles 10-11 and a readily, one-point recalibration in cycle 12.

No malfunctions were observed in the CURATE.AI algorithmic operations with 100 % (23/23) of the recommendations delivered on time. Data audit revealed one IgM data point (cycle 14) was incorrectly provided to the CURATE.AI team and needed to be retrospectively adjusted from 59.5 g/L to 59.8 g/L. CURATE.AI recommendation was not affected. User learning curves were not evaluated, and no preclinical studies were performed on the application of CURATE.AI to WM.

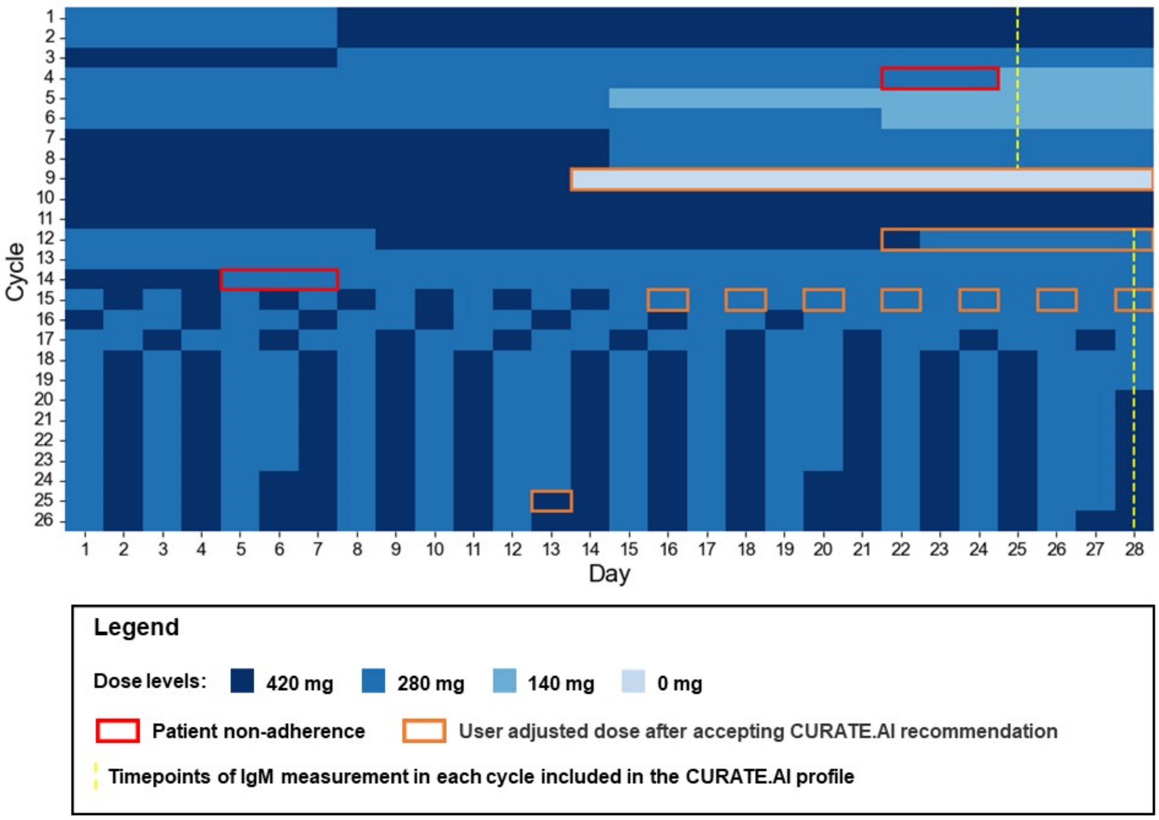

Supplementary Figure 1 | Daily effected Ibrutinib doses in each cycle based on pharmacovigilance information during the 26 cycle-long treatment. In the unusual patient non-adherence event the patient took a daily dose of 420 mg instead of 280 mg for 3 days. The user adjusted the dose after accepting CURATE.AI recommendation in cycle 9 (due to COVID-19 onset), cycles 12 and 14 (due to toxicities) and cycle 25 (higher MTD; counted as rejecting CURATE.AI dose recommendation for the cycle).

Supplementary Table 1 | PRECISE CURATE.AI Outcome Measures for Cohort WM (BTK Inhibitors in Waldenström Macroglobulinaemia)

Outcome Measures	Green	Yellow	Red
Primary Outcome Measures			
CURATE.AI applicability: percentage of participants in whom we successfully apply CURATE.AI profile.<br><br>A decision on whether we "successfully apply" the CURATE.AI profile requires expert judgement and cannot be made based on a purely numerical process. The expert panel will consider the following factors with careful regard for the individual circumstances of each participant:<br>1. Error/variance (biological/analytical) is sufficiently small to allow accurate predictions<br>2. Profile can be generated sufficiently early for the participant to potentially benefit;<br>3. Dose-dependent relationship is observed;<br>4. Profile is actionable (i.e. fulfils the co-investigator's pre-specified safety requirements);<br>5. Systemic changes in the participant which require profile recalibration are rare or readily assimilated into the CURATE.AI algorithm.	>70%	10-70%	<10%
Secondary Outcome Measures			
Patient adherence: percentage of participants who always adhered to the prescribed dose whenever they took their medication, as measured by the standardized pharmacovigilance protocol.	>90%	10-90%	<10%
Timely delivery of CURATE.AI recommendations to the clinician: percentage of CURATE.AI recommendations provided in time for the next chemotherapy cycle, across all participants and cycles.	100%	10-99%	<10%
CURATE.AI relevance: percentage of dosing events across all participants and cycles in which CURATE.AI recommendation is considered in the clinical decision-making process	>70%	10-70%	<10%
Clinician adherence: percentage of CURATE.AI recommended doses that were used by the co-investigator.	>70%	10-70%	<10%
Clinically significant dose changes: percentage of participants in whom the CURATE.AI-guided cumulative dose is substantially (≥10%) different from the projected standard-of-care cumulative dose, which is defined as the maximum dose of the modulated drug* multiplied by the number of completed chemotherapy cycles.	>20%	1-20%	0%

Supplementary Table 2 | Changes made to the protocol after recruitment of the first patient in PRECISE CURATE.AI pilot study relevant to WM cohort (BTK Inhibitors in Waldenström Macroglobulinaemia (WM)). sFLC – serum-free light chain

Protocol version	Section change was made to	Changes	Reason
Version 4 (29-Oct-2020) Approved on 24-Dec-2020	Background and rationale	Addition of WM cohort background and rationale.	Expand trial to include the WM cohort
	Hypothesis and objectives	Addition of WM cohort hypothesis, objectives and outcome measures.	
	Study population	Addition of WM cohort enrolment, inclusion and exclusion criteria.	
	Trial schedule	Addition of WM cohort trial schedule (visits, examinations, procedures, etc).	
	Study design	Addition of WM cohort specifics to study design.	
	Methods and assessment	Addition of WM cohort visits and procedures	
	Trial materials	Addition of WM cohort single agent ibrutinib and sFLC.	
	Treatment	Addition of WM cohort single agent ibrutinib dose.	
	Sample size and statistical methods	Addition of WM cohort statistical and analytical plans determination of sample size.	
Version 5 (10-Mar-2021) Approved on 30-Apr-2021	Background and rationale, study design, methods, and assessments	Possibility of including administered doses, corresponding phenotypic responses, and treatment-related toxicities (optional) in CURATE.AI processes for participants who started on the selected therapy prior to their engagement with the study.	Enhance CURATE.AI usability
	Hypothesis and objectives	New definition and assessment guidelines for CURATE.AI relevance.	Improve feasibility assessment
	Study population	Introduction of specific inclusion criterium for WM cohort: if patients have already started treatment on this regimen, they may still be eligible to enrol, provided they fulfil all other criteria and approval is sought by PI and Sponsor.	Facilitate enrolment
	Trial schedule	Clarification for WM cohort: the blood draws will be performed by trained personnel and may include home phlebotomist.	Clarity
	Study design	Allowing the collection of patient’s previously recorded treatment data up till 6 months from trial engagement, at the approval of the principal investigator, to be included in the CURATE.AI process.	Contextual information based on past events
Version 6 (2-Aug-2021) Approved on 19-Aug-2021	Trial schedule, study design	Conducting patient survival follow-up via telephone every 6 months for 3 years after completion of the trial.	Survival follow-up
	Methods and assessment	Categorizing and specifying procedures performed as part of standard-of-care or only for research purposes.	Clarity
Version 8 (5-May-2022) Approved on 19-May-2022	Inclusion criteria	Restricting recruitment to Singapore and Singapore Permanent Residents. Non-residents can be recruited only at the approval of the principal investigator and sponsor.	Trial Practicality
Version 10 (3-Oct-2022) Approved on 19-Oct-2022	Background and rationale, hypothesis and objectives, methods and assessment	Inclusion of sFLC as a primary marker.	Expand trial to test a new marker
	Trial schedule	Clinician being able to select either IgM or sFLC for a specific patient to guide dosing with CURATE.AI.	Expand trial to test a new marker
	Study design	Communications between the CURATE.AI team, trial coordinators and clinicians may be managed through an engagement platform, (E.g. BotMD Care dashboard).	Enhance data flow and safety
	Ethical considerations	Details on sample retention and use of leftover samples in future research	Regulatory requirement
Version 12 (31-Jul-2023) Approved on 26-Aug-2023	Background and rationale, trial schedule, Treatment	Stable disease management is defined as CURATE.AI providing dose recommendations with the objective to keep the patient's disease stable.	Enable CURATE.AI engagement to align with stable disease management
	Background and rationale, trial schedule, treatment	1. Addition of a new BTK inhibitor drug, acalabrutinib.<br>a. Maximum total cumulative dose per cycle of acalabrutinib in the predetermined safety range is set at 100% of standard starting dose (i.e. 100 mg twice daily for 4 weeks, which constitutes the total of 5600 mg per cycle).<br>b. Acalabrutinib is safe at a daily dose of 200 mg and below, and it must not exceed the daily limit of 200 mg.<br>2. The updated regimens are:<br>a. Switching from Ibrutinib to Acalabrutinib or vice versa during treatment.	Expand trial to include an additional regimen

		b. Single-agent BTK inhibitor (either Ibrutinib or Acalabrutinib) administration from the start of treatment.	

Supplementary Table 3 | Patient data types used for CURATE.AI operations. Only patient data are used for the generation of the patient profile. Clinical Research Coordinator (CRC) anonymizes the data before their transfer to the CURATE.AI team via an email attachment. EHR – electronic health record.

**Data Type**	**Data Details**<br><br>***[original source]***	**Reason for collection and additional information on pre-processing and handling of missing/low-quality data if relevant**
Scheduling Information	Date of the patient clinical visit, examinations and tests<br><br>*[laboratory test documentation]*	Scheduling information is used to schedule CURATE.AI operations for timely delivery of the dose recommendation to the user, as well as to verify how well the monitoring data overlap with the schedule of each cycle.
	Date of the cycle's start and end<br><br>*[hospital triaging data]*	
BTK Inhibitor Dose and Dose Selection Range	Prescribed dose of BTK Inhibitor – ibrutinib or Acalabrutinib – represented as a total amount per cycle.<br><br>*[trial dedicated EHR]*	CURATE.AI uses Ibrutinib effected dose as one of its inputs in the [dose:response] data pair. It is calculated by adjusting the prescribed dose by the patient adherence. If no information on patient adherence is provided, 100% adherence is assumed.
	Patient adherence<br><br>*[pharmacovigilance performed under the trial]*	
	Restrictions to the dose selection range<br><br>*[treating clinician]*	User may adjust dose selection range from 50-100% to suit patient current circumstance. If no information is provided and there is no response to the CURATE.AI team email enquiry, the dose selection range as in the prior cycle is assumed.
Phenotypic Response	Response biomarker – IgM or sFLC – level at the start and end of each cycle<br><br>*[laboratory test documentation]*	CURATE.AI uses selected response biomarker as one of its inputs in the [dose:response] data pair. An absolute value is used in the algorithmic processes.
Blood Biomarkers Monitoring	Full blood count, renal panel, liver panel, M band, IgM or sFLC (depending what is used as the phenotypic response for the given patient)<br><br>*[laboratory test documentation]*	The blood biomarkers point to the clinical outcomes and any potential systemic changes that the patient may be experiencing that could led to dose range restrictions or a recalibration. Not used directly in CURATE.AI algorithmic processes.
Safety	Details of adverse events and side effects including description, severity, duration, hospitalisation duration, likelihood of being related to BTK inhibitor, and whether they restrict dose selection range.<br><br>*[trial dedicated EHR]*	The adverse events and side effects inform on 1) potential for restriction of the dose selection, 2) ongoing systemic changes, 3) expected dose interruptions. In the luck of information on safety, no issues are assumed.
Concomitant Drugs	Changes in concomitant drugs<br><br>*[trial dedicated EHR]*	Concomitant drugs may interact with the patient response to the treatment. Sustained changes in the concomitant drugs may lead to the need for recalibration.

Supplementary File 1

STUDY PROTOCOL

Personalised, Rational, Efficacy-driven Cancer drug dosing via an artificial Intelligence SystEm – CURATE.AI (PRECISE CURATE.AI trial).

The protocol below is focused on the Waldenström Macroglobulinemia cohort and is a supplementary file for **Personalized dose selection for the first Waldenström Macroglobulinemia patient on the PRECISE CURATE.AI trial** manuscript**.**

TABLE OF CONTENTS

1. BACKGROUND AND RATIONALE 4
   1.1. General Introduction 5
   1.2. Rationale and justification for the Study 5
   A. Rationale for Doses Selected 5
   B. Rationale for Study Population 6
   C. Rationale for Study Design 7
2. HYPOTHESIS AND OBJECTIVES 8
   2.1. Hypothesis 8
   2.2. Primary Objectives 8
   2.3. Secondary Objectives 8
   2.4. Exploratory Objectives 8
   2.5. Potential Risks and Benefits - endpoints 9
3. STUDY POPULATION 11
   3.1. List the number of subjects to be enrolled. 11
   3.2. Criteria for Recruitment 11
   A. Inclusion Criteria 11
   B. Exclusion Criteria 12
   3.3. Withdrawal Criteria 12
   3.4. Subject Replacement 13
4. TRIAL SCHEDULE 13
5. STUDY DESIGN 14
   5.5. CURATE.AI Internal Workflow 15
   5.6. Summary of Study Design 19
6. METHODS AND ASSESSMENTS 19
   6.1. Randomisation and Blinding 19
   6.2. Contraception and Pregnancy Testing 19
   6.3. Study Visits and Procedures 19
      A. Screening Visits and Procedures 19
      B. Follow-up Visits and Procedures 20
7. TRIAL MATERIALS 21
   7.1. Trial Product (s) 21
   7.2. Storage and Drug Accountability 21
8. TREATMENT 21
   8.1. Rationale for Selection of Dose 21
   8.2. Study Drug Formulations 22
   8.3. Study Drug Administration 23
   8.4. Specific Restrictions / Requirements 23
   8.5. Blinding 23

8.6. Concomitant therapy 23
8.7. Treatment interruption 23

9. SAFETY MEASUREMENTS 23

9.1. Definitions 23
9.2. Collecting, Recording and Reporting of "Unanticipated Problems Involving Risk to Subjects or Others" – UPIRTSO events to the NHG Domain Specific Review Boards (DSRB) 24
9.3. Collecting, Recording and Reporting of Serious Adverse Events (SAEs) to the Health Science Authority (HSA) 25
9.4. Safety Monitoring Plan 26
9.5. Complaint Handling 26

10. DATA ANALYSIS 26

10.1. Data Quality Assurance 26
10.2. Data Entry and Storage 26

11. SAMPLE SIZE AND STATISTICAL METHODS 27

11.1. Determination of Sample Size 27
11.2. Statistical and Analytical Plans 27

12. ETHICAL CONSIDERATIONS 28

12.1. Informed Consent 28
12.2. IRB review 28
12.3. Confidentiality of Data and Patient Records 28

13. PUBLICATIONS 29

14. RETENTION OF TRIAL DOCUMENTS 29

REFERENCES: 31

STUDY PROTOCOL

1. BACKGROUND AND RATIONALE

Cancer patients are given drug combinations that promote cancer cell elimination. The final drug concentration in the body must fall within a narrow range that maximises cancer elimination while minimizing toxic side effects. The complexity of this task increases significantly with the number of drugs given in combination due to increasing parameters and stochastic behaviour of a biological system. Currently, the established approach is to select maximum tolerated doses (MTD) – the highest drug doses that do not cause unacceptable side effects[1-3]. Treatment efficacy does not guide dose selection. Combined with limited personalisation, this dosing strategy often results in sub-optimal outcomes of the treatment.

CURATE.AI is an AI-derived, mechanism-independent, small data technology platform for personalised, dynamic dosing. CURATE.AI uses a quadratic equation to generate individualised CURATE.AI profile and dosing recommendation based on only that individual's medical data: drug doses and the corresponding response marker (e.g. blood tumour markers). Profile recalibration via CURATE.AI facilitates dynamic dosing and personalised care throughout the treatment duration, aimed at achieving the highest efficacy within pre-specified safe dose ranges.

CURATE.AI is an indication-agnostic platform that has already been applied clinically for a range of indications including in oncology. CURATE.AI can be applied to indications that demonstrate regularly measured dose-dependent relationship between the treatment dose and the treatment response through response markers (i.e. efficacy or toxicity marker level). Blood-based response markers are one of the most suitable markers to be used in CURATE.AI as they allow for more frequent quantification of response and are less invasive.

Waldenstrom Macroglobulinemia (WM) is an uncommon condition characterized by the excessive production of monoclonal immunoglobulin M (IgM). The global age-adjusted incidence rate for this disease is 0.42 per 100,000 person-years. Current standardized dosing methods often result in inconsistent efficacy, unwanted side effects, and increased costs. Traditional personalized approaches, such as dose adjustment (titration), frequently prove inadequate for optimizing treatment.

This pilot study aims to set foundation to investigate the applicability of the CURATE.AI platform for WM within the current clinical setting for guided dosing of Bruton's tyrosine kinase (BTK) inhibitors.

Individualised CURATE.AI profiles will be generated based on each participant's response to a set of drug doses. Subsequently, the personalised CURATE.AI profile will be used to recommend the efficacy-driven dose, and stable disease management dose. Patients with stable disease have a disease that is neither increasing or decreasing in severity or extent[4]. Stable disease management is defined as CURATE.AI providing dose recommendations with the objective to keep the patient's disease stable. CURATE.AI will operate only within the safety range for each drug pre-specified for each participant.

This pilot study will inform the investigators on the logistical and scientific feasibility of performing a randomised study with the selected systemic therapy regimen and response marker. A secondary objective is to collect toxicity and efficacy data using established and exploratory response markers within and in-between cycles as exploratory outcomes. Additionally, CURATE.AI may allow to glean into the costs of personalized dosing strategy through an analysis toward economic evaluation to better understand the potential financial implications of adopting CURATE.AI in clinical settings that may affect RCT trial design to best align with logistical and implementation considerations.

1.1. General Introduction

As the optimal drug doses for commonly used systemic therapy regimens are not well defined, particularly in combination drug regimens, physicians rely on their experience and the population-based dosing guidelines. Patients' responses to the selected doses vary greatly due to individual characteristics of each patient including their disease states, genetics and drug-drug interactions[5]. As a result, patients may experience overdosing and underdosing events, which respectively may cause toxicities and reduced efficacy.

Traditional data-driven approaches, such as statistical metamodeling and pharmacokinetic- or pharmacodynamic-driven precision dosing, demand substantial volume of population information. The data analysis team involved in this study harnessed AI to discover that a disturbance in a complex system can be described with an equation. Specifically, the correlation of drug doses and the phenotypic response marker (in this oncology trial, it refers to blood response markers) in a human system can be represented with a quadratic equation. CURATE.AI – a small data, AI-derived technology platform based on this discovery – allows personalised guidance of an individual's dose modulations based only on that individual's data. Additionally, CURATE.AI is mechanism-independent, and dynamically adapts to changes experienced by the participant, providing dynamic dose optimisation throughout the duration of the participant's treatment.

CURATE.AI dosing recommendations are always within a range that is clinically safe as predetermined by the co-investigators. CURATE.AI recommends personalised doses that take into account the participant's systemic changes, which has a potential to dramatically improve efficacy and safety.

1.2. Rationale and justification for the Study

A. Rationale for Doses Selected

Drug dosing safety ranges are predefined by the co-investigators based on the population data and participant-specific circumstances.

The first stage of the trial aims to obtain a personalised CURATE.AI profile for each participant, based on their phenotypic response to a set of drug doses. The doses will be recommended by the CURATE.AI team, when relevant to the clinical decision-making process. Once an actionable profile is obtained, dose recommendations are based on the profile and aimed to treat the participant.

CURATE.AI dose recommendations will always be within:

1) The predetermined safety range of 50% to 100% of dose used in standard of care treatment with single agent ibrutinib.
2) Participant-specific dosing range (the dosing range accounting for the specific participant's personal medical history and clinical context) that might be different than the safety dose range used in standard of care treatment as specified by the co-investigators.

If no recommendations can be given that fulfils the above requirements, participant will be given the dose according to the standard of care. Co-investigators will have the final say on whether to use the dose recommended by CURATE.AI. The participant may receive dose adjustments beyond the recommendations provided by CURATE.AI, if the co-investigator deems it necessary. Subjected to the co-investigator's clinical judgement, dose recommendations by CURATE.AI to patients with stable disease will align with stable disease management. Patients with stable disease have a disease that is neither increasing nor decreasing in severity or extent[4]. Stable disease management is defined as CURATE.AI providing dose recommendations with the objective to keep the patient's disease stable.

In addition to the prescribed drug doses and the corresponding phenotypic responses, the participant's previously recorded data may be collected, only upon the approval of PI and be included in the CURATE.AI processes detailed in section 5.5. Specifically:

- For participants who started on their selected therapy prior to their engagement with the study, the previously recorded data, limited to: administered drugs, corresponding phenotypic responses and treatment-related toxicities (optional) to be included in the CURATE.AI processes.

- For participants who were not treated with the study therapy prior to their engagement with the study, the previously recorded data, limited to: administered drugs, corresponding longitudinal phenotypic response and treatment-related toxicities (optional) may be used to provide information on:

    - The status of the participants to an event encountered prior to engagement with the study, which the event / similar events are known to take place during the trial (e.g. Information on response markers during the last vaccination for an upcoming booster vaccination).
    - Possible fluctuations in response marker measurements resulting from similar events encountered during the trial.

The information gathered will inform on systemic changes for the participant, which will be incorporated as part of the CURATE.AI processes detailed in section 5.5.

B. Rationale for Study Population

The study cohort in this trial will comprise of participants diagnosed with WM undergoing or planned for treatment with ibrutinib. We chose ibrutinib as it is a potent BTK inhibitor that has revolutionised the treatment of B cell malignancies and shown favourable clinical efficacy as compared to other novel single agents and comparable efficacy as compared to combination therapies[6]. However, the current recommended dose of 420 mg has been found to be

inadequately tolerated, with a tendency towards required dose reductions and discontinuation[7]. In support of dose de-escalation, a recent pilot trial has shown that a low dose of 140 mg ibrutinib has an irreversible BTK inhibition and achieves a consistent pharmacodynamic effect of more than 95% of BTK receptors occupancy, without losing any biological activity[8]. Furthermore, as ibrutinib often has to be administered indefinitely in responders, with annual costs exceeding $130 000 in the United States, financial implication is a major concern[9]. To complicate matters, patients treated with ibrutinib often take concomitant medications that increase ibrutinib toxicities or decrease ibrutinib efficacy[10]. Yet, without definitive evidence from large-scale randomised trials and inability to reliably identify patients who may benefit from a lower dose of ibrutinib, clinicians are reluctant to implement dose reductions except in clear indications such as toxicity and drug-drug interactions (DDIs)[7]. In this backdrop of toxicity, cost and DDIs, patients may stand to benefit from CURATE.AI's efficacy-driven personalised dosing strategy. WM, an approved indication for ibrutinib, is a rare neoplasm whose burden can be quantified with blood-based markers, e.g., serum Immunoglobulin M (IgM) paraprotein levels and serum free light chain (sFLC), which CURATE.AI may use as inputs. Taken together, CURATE.AI is well positioned to provide much needed, guided, personalised dose adjustment for participants with WM on ibrutinib regimen.

In the same drug class as ibrutinib, acalabrutinib is the next-generation BTK inhibitor that has demonstrated non-inferior patient's survival outcome with lowered bleeding and cardiovascular adverse effects in the ELEVATE-RR trial (NCT02477696)[11]. Compared to ibrutinib, acalabrutinib appears to have a better patient safety profile[12]. The patient may undergo a regimen change between ibrutinib and acalabrutinib during the treatment, or be administered with a single-agent BTK inhibitor regimen from the start of treatment for WM.

CURATE.AI will facilitate personalised treatment to each of the participants by recommending optimal doses in a dynamic fashion. Criteria for recruitment allow a high variability in the participant population to reflect a true variability in the cases faced in the clinical practice.

C. Rationale for Study Design

In this pilot study, participants will undergo a stage of CURATE.AI profile generation and a stage of CURATE.AI profile-based, efficacy-driven drug dosing. As there are no prior clinical trials using CURATE.AI with the specific drug regimens for WM listed below, this pilot study will focus on the practicality and feasibility of using CURATE.AI in this clinical context. An interim analysis will be conducted using the data generated from these participants, which will include formal power and statistical sample size calculations. Based on these outcomes, we will consider cohort expansion or an RCT. Specifically, the interim analysis will aid the decisions on (1) whether to proceed with future randomised controlled trials; (2) their design (superiority, equivalence or non-inferiority); (3) logistical and practical aspects of running a large-scale randomised trial; (4) patient population selection for the randomised trial (5) potential applicability of CURATE.AI in a wider range of systemic therapy regimens, response markers and/or expansion of the current cohort to elicit further data on secondary endpoints and/or new randomized cohorts.

2. HYPOTHESIS AND OBJECTIVES

2.1. Hypothesis

The primary hypothesis of this study is that CURATE.AI will meet the feasibility criteria, set out below in sections 2.2 and 2.3 in the study protocol, for the selected systemic therapy regimens and response markers for the treatment of WM. Additional exploratory hypotheses are listed in section 2.4.

2.2. Primary Objectives

The primary objective of this study is to assess the logistical and scientific feasibility of an RCT for CURATE.AI-guided dosing with the selected systemic therapy regimens and response markers.

Specifically, logistical feasibility refers to:
(1) ability to provide timely dose recommendations by CURATE.AI;
(2) participant adherence to CURATE.AI-recommended doses;
(3) physician adherence to CURATE.AI-recommended doses.

Scientific feasibility is defined by the following questions:
(1) whether CURATE.AI profiles can be successfully created and applied;
(2) in which patient groups can these profiles be successfully applied;
(3) whether the CURATE.AI-recommended dose is substantially different from the standard-of-care dose.
(4) whether CURATE.AI is relevant under the current clinical practice

2.3. Secondary Objectives

1. Collect response marker levels change throughout the duration of the treatment for patients treated based on CURATE.AI guided dosing

2. Collect Safety and Toxicity throughout the duration of the treatment for patients treated based on CURATE.AI guided dosing

2.4. Exploratory Objectives

1. Evaluate the higher frequency of response marker (E.g. IgM and/or sFLC) measurements in response to modulated doses for information about temporal dynamics of tumour response to modulated doses in WM.

2. Explore the hypothesis that dose modulations with CURATE.AI will yield new information on WM.

3. Explore mass spectrometry quantified M-proteins for monitoring treatment response dynamics in comparison to standard-of-care derived response markers (E.g. IgM, sFLC or M-band levels).

4. Explore the utility of mass spectrometry quantified M-proteins as a response marker in serial measurements with modulated doses and as an input for CURATE.AI to generate dose recommendations. This analysis will not be used to prospectively guide dosing.

5. Explore the hypothesis that dynamic changes in mass spectrometry quantified M-proteins correlate with clinical outcomes for WM.

2.5. Potential Risks and Benefits - Endpoints:

The primary outcome measure is the percentage of participants in whom we successfully apply CURATE.AI profile, as concluded by expert judgement based on multiple factors listed in Table 1. This is the main outcome which we will use to judge the scientific feasibility of the RCT according to 'the traffic light system' defining progression criteria.

The secondary outcome measures focus on both scientific and logistical feasibility (Table 1)

The exploratory outcome measures mainly relate to efficacy and toxicity (Table 1).

Safety will be assessed as part of exploratory endpoints on the basis of the frequency and severity of adverse events. Adverse events are graded according to the National Cancer Institute's Common Terminology Criteria for Adverse Events Version 4.0. Safety assessments are based on reported adverse events, clinical laboratory tests (haematologic testing and serum chemical testing), vital signs, physical examinations and ECOG performance status.

Possible risks to the participant include: lower treatment efficacy due to dose adjustments (including possible dose reductions) and the risk of additional blood draws (pain, bleeding, bruising, swelling, fainting, infection, etc.). The potential toxicities stemming from the use of the experimental platform CURATE.AI for dose adjustment are the same as in standard of care, though their frequencies may vary (likely reduced) compared to standard of care. For a full explanation in layman language, please refer to Appendix 1.

Table 1. Outcome measures and progression criteria for the PRECISE CURATE.AI pilot study, based on 'the traffic light system': Green: a future randomised trial is definitely feasible. Yellow: a future randomised trial is possibly feasible if its design is appropriately modified. Red: a future randomised trial is unfeasible in its current form.

Abbreviations: CTCAE, Common Terminology Criteria for Adverse Events. sFLC, serum free light chains.

Outcome Measures	Green	Yellow	Red
Primary Outcome Measures			
CURATE.AI applicability: percentage of participants in whom we successfully apply CURATE.AI profile.<br><br>A decision on whether we "successfully apply" the CURATE.AI profile requires expert judgement and cannot be made based on a purely numerical process. The expert panel will consider the following factors with careful regard for the individual circumstances of each participant:<br>(1) Error/variance (biological/analytical) is sufficiently small to allow accurate predictions<br>(2) Profile can be generated sufficiently early for the participant to potentially benefit;<br>(3) Dose-dependent relationship is observed;<br>(4) Profile is actionable (i.e. fulfils the co-investigator's pre-specified safety requirements);<br>(5) Systemic changes in the participant which require profile recalibration are rare or readily assimilated into the CURATE.AI algorithm.	>70%	10-70%	<10%
Secondary Outcome Measures			
Patient adherence: percentage of participants who always adhered to the prescribed dose whenever they took their medication, as measured by the standardised pharmacovigilance protocol.	>90%	10-90%	<10%
Timely delivery of CURATE.AI recommendations to the clinician: percentage of CURATE.AI recommendations provided in time for the next chemotherapy cycle, across all participants and cycles.	100%	10-99%	<10%
CURATE.AI relevance: percentage of dosing events across all participants and cycles in which CURATE.AI recommendation is considered in the clinical decision-making process	>70%	10-70%	<10%
Physician adherence: percentage of CURATE.AI recommended doses that were used by the co-investigator.	>70%	10-70%	<10%
Clinically significant dose changes: percentage of participants in whom the CURATE.AI-guided cumulative dose is substantially (≥10%) different from the projected standard-of-care cumulative dose, which is defined as the maximum dose of the modulated drug* multiplied by the number of completed chemotherapy cycles.	>20%	1-20%	0%
Exploratory Outcome Measures			
Efficacy:<br>(1) Percentage of trial participants with clinical progressive disease (defined as the co-investigator deeming that the patient will not benefit any further from the chemotherapy regimen and considering stopping it) at the time of the first radiological assessment performed as per standard-of-care.	N.A.	N.A.	N.A.

(2) Temporal variation in response marker (E.g. IgM and/or sFLC) level from baseline to trial conclusion.<br>(3) Maximal reduction in response marker (E.g. IgM and/or sFLC) level measured as part of baseline investigations			
Toxicity: percentage of trial participants with clinically relevant toxicities of grades 3-4 based on CTCAE version 4.0.	N.A.	N.A.	N.A.
Data collection and explorative analysis of response markers (E.g. IgM and/or sFLC) in higher frequency serial measurements after modulated dosing in relation to standard frequency readings and other efficacy measures, e.g IWWM-8	N.A.	N.A.	N.A.
Data collection and explorative analysis of efficacy and safety measures when the dosing is guided by CURATE.AI.	N.A.	N.A.	N.A.
Data collection and exploratory analysis of mass spectrometry quantified M-proteins as:<br>(1) a response marker in serial measurements and after modulated dosing;<br>(2) potential input for CURATE.AI	N.A.	N.A.	N.A.
Data collection and explorative analysis of mass spectrometry quantified M-proteins in comparison to standard-of-care derived response markers (E.g. IgM, sFLC or M-band levels) for monitoring treatment response dynamics.	N.A.	N.A.	N.A.

*The maximum, standard-of-care dose of the modulated drug is:
1. Ibrutinib (single agent, 420 mg once daily)
2. Acalabrutinib (single agent, 100 mg twice daily)

3. STUDY POPULATION

3.1. List the number of subjects to be enrolled.

10 participants diagnosed with WM who are undergoing or starting on treatment with single agent BTK inhibitor will be enrolled. There are no restrictions based on race. Pregnant females are excluded from the study.

3.2. Criteria for Recruitment

Patients fulfilling the below inclusion and exclusion criteria will be enrolled as study participants. Criteria for recruitment allow a high variability in the participants to reflect a true variability in the cases faced in the clinical practice.

A. Inclusion Criteria

1. Males and females ≥ 21 years of age.

2. Singaporeans and Singapore Permanent Residents only. Non-residents can be recruited only at the approval of PI and Sponsor.
3. Eastern Cooperative Oncology Group (ECOG) Performance Status of 0 to 2.
4. Patients must meet the following clinical laboratory criteria within 21 days of starting treatments:
    a) Absolute neutrophil count (ANC) ≥ 1,000/mm^3 and platelet ≥ 50,000/mm^3
    b) Total bilirubin ≤ 1.5 x the upper limit of the normal range (ULN). Alanine aminotransferase (ALT) and aspartate aminotransferase (AST) ≤ 3 x ULN of ≤ 5 ULN if involvement of the liver.
    c) Calculated creatinine clearance ≥ 30 mL/min or creatinine < 1.5 x ULN.
5. WM as defined by the World Health Organisation 2016 diagnostic criteria.
6. Treatment with BTK inhibitor. If patients have already started treatment on this regimen, they may still be eligible to enrol, provided they fulfil all other criteria and approval is sought by PI and Sponsor
7. Immunofixation confirms immunoglobulin M paraprotein and total IgM > 2 x ULN.

B. Exclusion Criteria

1. Patients who are lactating or pregnant.
2. Patients with clinically significant hypersensitivity to one or more of the selected regimen's constituent drug(s)
3. Contraindication to any of the required concomitant drugs or supportive treatments.
4. Any clinically significant medical disease or psychiatric condition that, in the co-investigator's opinion, may interfere with protocol adherence or a subject's ability to give informed consent.
5. Systemic anti-lymphoma therapy within 3 weeks of enrolment. Steroids at a dose equivalent of prednisolone 30mg per day are allowed provided this is discontinued 72 hours prior to commencement of drug dosing on trial.
6. Need to withhold rituximab in view of the risk of IgM flare (applies to patients treated with rituximab-based regimens and with IgM as a response marker).
7. Platelet transfusion within 7 days of screening.
8. Granulocyte colony stimulating factor within 7 days of screening.

3.3. Withdrawal Criteria

A participant may be withdrawn from the study for any of the following reasons:

- Lost to follow-up
- Withdrawal by participant
- Disease progression
- Toxicity
- Death
- Other

The consequence of study withdrawal is that no new information will be collected from the withdrawn participant and added to the existing data or any database; however, every effort will be made to follow up all participants for safety.

3.4. Subject Replacement

Participants will be considered evaluable for primary end-point only if they are able to complete the first 2 cycles of systemic therapy during the initial treatment period post-recruitment [CURATE.AI profile calibration]. If a participant is unable to complete the first two cycles (uninterrupted without doses being held for reasons such as toxicity), the study team will continue monitor the participant for the duration of the trial for the safety endpoints. Unevaluable participant will be replaced with newly recruited participant. Also in the event that the patient, based on the clinicians assessment, meets any of the stopping rules and/or is moved to another regimen, depending on the trial stage, the patient will be considered for replacement, but will not have to be replaced (e.g. if the duration of the patient engagement with the trial has already provided enough data points to contribute to primary end-point assessment, then the patient will not be replaced).

4. TRIAL SCHEDULE

Participants will be followed up for the duration of their treatment with BTK inhibitor for 12 months. At the 12-month mark, a decision will be made by the principal investigator in consultation with the study sponsors and treating physician on whether to continue patient engagement in the study, likely at a lower frequency of follow-up, given that BTK inhibitor can often be dosed indefinitely until disease progression. After completing the study, the study team will perform survival follow up via telephone every 6 months for 3 years.

While both established response markers (E.g. IgM and sFLC) can be collected, treating physician will select which of them (E.g. IgM or sFLC) will be used for the specific patient to guide dosing with CURATE.AI. Efficacy and toxicity measurements at the end of each four-week cycle, together with an information on given drugs and their doses, and other patient data, will be used by CURATE.AI to suggest the recommended daily dosing of BTK inhibitor for the next cycle. It will also be used by co-investigators to make their decisions on whether or not to accept CURATE.AI's recommendations.

Other blood tests will be performed according to standard of care, usually at the end of each therapy cycle.

The standard drug dosing schedule of single-agent BTK inhibitor will be adhered to. The total cumulative dose of BTK inhibitor per cycle will be modulated by CURATE.AI. BTK inhibitor dose may vary on a daily basis.

Additional weekly blood draws to collect response markers (E.g. IgM and/or sFLC), as selected by the treating physician, solely for the purpose of research, will be performed in cycles 1 to 2 and once every two weeks in cycles 3 to 6. Optional blood draws in the remaining cycles will be limited to 1 draw. The blood draws will be performed by trained personnel and may include home phlebotomist.

Residual blood samples that are in excess from standard-of-care clinical tests, diagnostics and weekly analyses will be analysed for M-proteins with mass spectrometry.

See Fig. 1 below for the overall trial schedule.

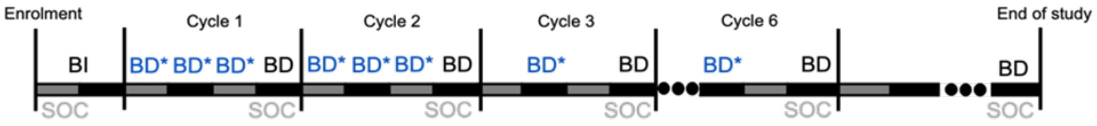

Fig. 1. Overall trial schedule for four weeklong cycles. BI: Baseline investigations as per standard-of-care, including collection of demographics, medical/treatment history, vital signs, and conducting complete physical examination including performance status evaluation, blood tests as clinically required. BD: Mandatory blood draws done every four weeks for the measurement of response marker(s) one week (preferably 3 days) before the start of each cycle. BD*: Additional blood draws for measurements of response marker(s), done once weekly for cycles 1-2 and once every two weeks for cycle 3-6 of treatment, performed solely for the purposes of the trial. Additional blood draws in subsequent cycles will be limited to one draw, if at all. SOC: Other tests performed as per standard-of-care. Residual blood samples that are in excess from standard-of-care clinical tests, diagnostics and weekly analyses will be analysed for M-proteins with mass spectrometry.

5. STUDY DESIGN

This is a multi-centre, one-arm, prospective pilot study of participants diagnosed with haematological neoplasm. The participants will be enrolled for the duration of their treatment with the selected regimen, as explained in Section 4. At the 12-month mark, the decision on the patient engagement with the study will be made by the principal investigator in consultation with the study sponsors and treating physician. After completing the study, the study team will perform survival follow up via telephone every 6 months for 3 years.

The CURATE.AI recommendations of total cumulative dose per cycle will be kept within the predetermined safety range. Co-investigators will have the final say on whether to use the dose recommended by CURATE.AI. Blood tests will be performed as per standard of care.

Dosing cycle and cycle duration remain unchanged. During the following events, dosing schedule may be adjusted to maintain the total cumulative dose per cycle:

a. A period of the cycle ending / begins on a holiday stretch
   Dosing schedule to take reference from CxD1. If dosing schedule falls on period of absence, approval may be sought from the principal investigator for an earlier or later dosing date.

b. Predetermined dosing events falling on a holiday
   Approval may be sought from the principal investigator for an earlier or later dosing date.

In the event of treatment interruption lasting beyond 28 days, there will be a case-review discussion meeting with the principal investigator.

Communications between the CURATE.AI team, trial coordinators and clinicians may be managed through an engagement platform, (E.g. BotMD Care dashboard) while complying with regulations related to confidentiality of the items discussed. Other established communication platforms could also be used alternatively.

Participants will receive the treatment in four-week cycles. The total cumulative dose of BTK inhibitor will be modulated with CURATE.AI, based on measurements of the response marker (E.g. IgM, sFLC).

Response marker measurements at higher frequency will be analysed to inform about the temporal dynamics of tumour response to modulated dosing.

Left-over serum remaining from the blood samples after standard-of-care clinical testing and weekly analyses following the trial schedule will be analysed for M-proteins with mass spectrometry.

5.1. CURATE.AI Internal Workflow

CURATE.AI operations are specified as follows (the study workflow is shown in Fig.2 below). First, the participants will enter CURATE.AI data collection stage for CURATE.AI profile generation. For CURATE.AI profile generation, a minimum of 3 dose levels and corresponding response marker readouts are needed for the modulated drug. After obtaining the first response marker readout a set of potential doses to pick from will be recommended by the CURATE.AI team with calibration-intent, based on the mathematical requirements of the method.

In addition to the prescribed drug doses and the corresponding phenotypic responses, the participant's previously recorded data may be collected, only upon the approval of PI and be included in the CURATE.AI processes. Specifically:

- For participants who started on the study therapy prior to their engagement with the study, the previously recorded data, limited to: administered drugs, corresponding phenotypic responses and treatment-related toxicities (optional) to be included in the CURATE.AI processes.

- For participants who were not treated with the study therapy prior to their engagement with the study, the previously recorded data, limited to: administered drugs, corresponding longitudinal phenotypic response and treatment-related toxicities (optional) may be used to provide information on:

    - The status of the participants to an event encountered prior to engagement with the study, which the event / similar events are known to take place during the trial (e.g. Information on response markers during the last vaccination for an upcoming booster vaccination).
    - Possible fluctuations in response marker measurements resulting from similar events encountered during the trial.

The information gathered will inform on systemic changes for the participant, which will be incorporated as part of the CURATE.AI processes. The data extracted from the records would be confined to 6 months prior to patient's trial engagement. The database being accessed for data collection will be EPIC. The type of data collected would be administered drugs, corresponding phenotypic responses, treatment-related toxicities, and covid/vaccination history.

The dose recommendations will be within 1) predetermined safety range and 2) participant -specific dosing range (the dosing range accounting for the specific participant's personal medical history and clinical context) that might be different than the safety dose range used in standard of care treatment as specified by the co-investigator. The predetermined safety ranges are 50% to 100% of dose used in

standard of care treatment with single agent ibrutinib, or ⩽100% of dose used in standard of care treatment with single agent acalabrutinib.

If no recommendation can be given that fulfils the above requirements, participant will be given the treatment dose according to the standard of care. Co-investigators will have the final say on whether to use the dose recommended by CURATE.AI. The participant may receive dose adjustments beyond the recommendations provided by CURATE.AI, if the co-investigators deem it necessary. For the participants who have started on the selected therapy prior to their engagement with the study, pre-existing data limited to: drug doses corresponding response marker readouts and treatment related toxicities (optional) may be included in CURATE.AI processes.

Subjected to the co-investigator's clinical judgement, patients may undergo a regimen change from ibrutinib to acalabrutinib (or vice versa) during the treatment, or be administered with a single-agent BTK inhibitor regimen from the start of treatment. Addition or removal of drugs may lead to recalibration of the patient's CURATE.AI profile, which evolves with the patient's treatment.

Once 3 valid data pairs (3 dose modulations with the 3 corresponding response marker readouts) are obtained, the data set will be checked for dose-dependent response. If the modulations do not yield a dose-dependent response (e.g. response marker readouts were affected by the factors unrelated to drug dose modulation) a new data pair will have to be acquired according to calibration-intent recommendation from the CURATE.AI team.

When dose-dependent response is obtained, the profile will be checked for actionability – an ability to recommend an optimal dose within the pre-specified personal safety range. Participants with actionable CURATE.AI profiles enter CURATE.AI efficacy-driven recommendation stage, where drug doses can be recommended by the team to the co-investigator, with a therapeutic intent, or stable disease management. A dynamic dose recommendation will be generated based on the patient's profile before every subsequent cycle of systemic therapy. If the modulation does not yield a dose-dependent response, and in the absence of systemic changes in the participant, a new data point will have to be acquired, and a new dose recommendation will be provided. If there are systemic changes in the participant, the profile will be recalibrated. During recalibration, doses for the patient will be selected on the basis of previous correlations. Actionable recommendations will be given until the end of the line of treatment. Patients with stable disease have a disease that is neither increasing nor decreasing in severity or extent[4]. Stable disease management refers to providing continual care and treatment to the patients with stable disease. In the context of CURATE.AI, stable disease management is defined as CURATE.AI providing dose recommendations with the objective to keep the patient's disease stable.

The dose recommendations will be within 1) population specific dosing range and 2) participants specific dosing range (the dosing range accounting for the individual circumstances of the specific participant).

Should the participants experience clinically relevant grade 3 or 4 non-haematological toxicity at a particular dose, the next dose recommendation by CURATE.AI will be restricted to a dose lower than the preceding dose (i.e. the recommended CURATE.AI dose will not be the same or higher than the dose the toxicity was experienced at). Co-investigators may also recommend similar limits to CURATE.AI predictions if the patient has clinically significant grade 3 or 4 prolonged haematological toxicities or prolonged grade 2 non-haematological toxicities.

When the co-investigator chooses not to use the recommendation the participant will be given the dose according to standard of care.

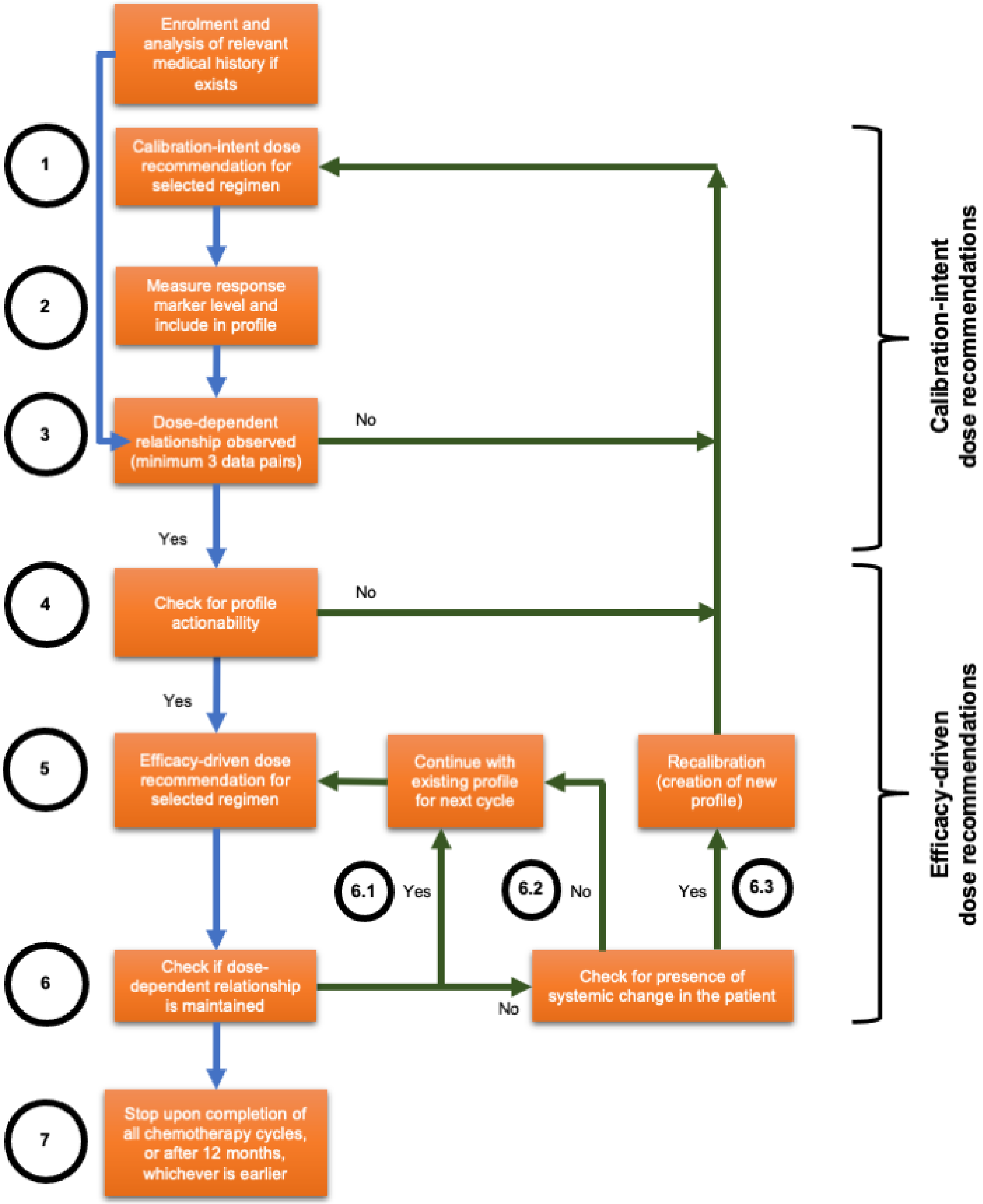

Fig. 2. CURATE.AI internal workflow for optimising therapy modulation with CURATE.AI, including scenarios that may lead to recalibration.

5.2. Summary of Study Design

This is a multi-centre, one-arm, prospective pilot study of participants diagnosed with haematological neoplasm. The participants will be enrolled for the duration of their treatment with the selected regimen, up to a maximum duration of 12 months, unless decided differently. A case review meeting with the principal investigator will be held in the event of patient experiencing treatment interruption longer than 28 days. After completing the study, the study team will perform survival follow up via telephone every 6 months for 3 years.

Personalised CURATE.AI profiles will be generated for each participant from their own data (treatment drug doses, response markers) and, for the cases when CURATE.AI can provide efficacy-driven dosing recommendations within the participant-specific safe dosing range, the profiles will be used for efficacy-driven guided dosing.

The CURATE.AI dose recommendations will be kept within the predetermined safety range. The co-investigator will have the final say on whether to use the dose recommended by CURATE.AI.

6. METHODS AND ASSESSMENTS

Patients who fulfil the inclusion and exclusion criteria will be prospectively recruited as participants. Baseline clinical and laboratory data will be collected prior to trial commencement. Participants will be monitored for toxicity and treatment efficacy. These will be recorded in the case report forms.

6.1. Randomisation and Blinding

No randomisation or blinding procedures are applicable to the study participants.
Co-investigators will not be blinded with respect to the intent of the CURATE.AI dose recommendations (profile generation driven or efficacy driven).

6.2. Contraception and Pregnancy Testing

The effects of proposed regimen on the developing human foetus at the recommended therapeutic dose are unknown. For this reason and because anti-tumour agents as well as other therapeutic agents used in this trial are known to be teratogenic, women of child-bearing potential and men must agree to use adequate contraception (hormonal or barrier method of birth control; abstinence) prior to study entry and for the duration of study participation. Should a woman become pregnant or suspect she is pregnant while participating in this study, she should inform her co-investigator immediately.

6.3. Study Visits and Procedures

A. Screening Visits and Procedures

Eligible patients will be asked for informed consent, and their baseline investigation and eligibility (inclusion and exclusion criteria) will be documented. For the participants who have started on the selected therapy prior to their engagement with the study, pre-existing data limited to: drug doses, corresponding response marker readouts and toxicities (optional) will be also

documented. The baseline investigations will be performed as per standard of care. The investigations may include the following:

- Demographics (age, sex, etc.)
- Medical/treatment history
- Complete physical exam including performance status
- Vital signs

Performed as per SOC

- Haematology, serum chemistries (full blood count, renal panel, liver function, LDH, uric acid, phosphate, calcium, etc.)
- Serum immunofixation
- Serum electrophoresis
- Response marker -E.g. IgM, sFLC (collected at SOC frequency)
- M band quantification
- Hepatitis B, C, HIV serology
- Glucose-6-phosphate dehydrogenase (G6PD) assay.

Performed for research purposes only

- Response marker – E.g. IgM, sFLC (collected during additional blood draws)
- Experimental response marker – mass spectrometry quantified M-proteins (from residual blood samples drawn during SOC procedures and during additional blood draws in excess from standard-of-care clinical tests, diagnostics and weekly analyses)

Response marker results from within 1 week of the start of the therapy and other results from within 30 days of the consent can be used as baseline results.

B. Follow-up Visits and Procedures

Within one week (preferably three days) before each subsequent cycle, the following tests should be performed, and data collected: haematology, serum chemistries, response markers and experimental response markers. Imaging should be repeated as per clinical need. In addition, physical examination including ECOG documentation, vital signs, adverse events documentation, and concomitant medications will be performed.

Please note that participants enrolled in the study may undergo blood tests more frequently than standard of care.

Participants will have a disease assessment done concurrently with each weekly or every two weeks visit for treatment. Other blood tests may be performed as per co-investigator's judgement. The co-investigator will perform history and physical examination, as well as review

of the adverse events prior to the start of every subsequent cycle of systemic therapy, as per local institution standards.

7. TRIAL MATERIALS

No placebo or control products are involved in the trial.

CURATE.AI in this context refers to CURATE.AI platform (software), expertise in operating CURATE.AI platform, drug dose recommendations generated by CURATE.AI platform and the accompanying analyses of the provided clinical data.

Only single agent BTK inhibitor will be used. Drug dosing schedules will be as per standard of care, with dose varied at maximum frequency of daily basis.

Laboratory analysis of response markers will be conducted as per institutional guidelines.

7.1. Trial Product (s)

Therapeutic agents that may be employed in this study include: single agent BTK inhibitor.

All drugs will be purchased from the local distributor and administered in an administration route according to institution standards of care.

7.2. Storage and Drug Accountability

The drugs used as part of the abovementioned systemic therapy regimens will be subjected to the same storage and accountability conditions as per institutional requirements utilised in standard clinical settings.

8. TREATMENT

8.1. Rationale for Selection of Dose

Throughout the course of the trial CURATE.AI dose recommendations will always be within 1) the predetermined safety range for the stuy and 2) participant-specific dosing range (the dosing range accounting for the specific participant's personal medical history and clinical context). Taken together, these form the participant-specific safe dosing range.

Maximum total cumulative dose per cycle of ibrutinib in the predetermined safety range is set at 100% of standard starting dose (i.e. 420 mg once daily for 4 weeks, which constitutes the total of 11760 mg per cycle). Minimum total cumulative dose per cycle of ibrutinib in the predetermined safety range is set at 50% of the standard starting dose (i.e. 280 mg once daily on alternative days with 140 mg once daily, which constitutes the total of 5880 mg per cycle). Maximum total cumulative dose per cycle of acalabrutinib in the predetermined safety range is set at 100% of standard starting dose (i.e. 100 mg

twice daily for 4 weeks, which constitutes the total of 5600 mg per cycle). Acalabrutinib is safe at a daily dose of 200 mg and below, and it must not exceed the daily limit of 200 mg). The dosing range may be altered to suit the specific circumstances of the participant, thus giving the participant-specific safe dosing range. Subjected to the co-investigator's clinical judgement, patients may undergo a regimen change from ibrutinib to acalabrutinib (or vice versa) during the treatment, or a single-agent BTK inhibitor will be administered as a single-agent regimen from the start of treatment. Changes in the choice of BTK inhibitors will be made at the discretion of the treating physician based on clinical criteria including lack of response or toxicity.

CURATE.AI recommendations for the daily dose and the total cumulative dose per cycle of the modulated drug will be generated prior to the start of each cycle. The CURATE.AI dose recommendations will be kept within the participant-specific safe dosing range. Co-investigator will have the final say on whether to use the dose recommended by CURATE.AI (i.e. the recommended CURATE.AI dose will not be the same or higher than the dose the toxicity was experienced at). Co-investigators may also recommend similar limits to CURATE.AI predictions if the patient has clinically significant grade 3 or 4 prolonged haematological toxicities or prolonged grade 2 non-haematological toxicities.

Should the participant experience clinically relevant grade 3 or 4 non-haematological toxicity at a particular dose, the next dose recommendation by CURATE.AI will be restricted to a dose lower than the preceding dose.

Should the participants achieve stable disease, according to the co-investigator's clinical judgement, dose recommendations by CURATE.AI will align with stable disease management. Patients with stable disease have a disease that is neither increasing nor decreasing in severity or extent[4]. Stable disease management is defined as CURATE.AI providing dose recommendations with the objective to keep the patient's disease stable.

The CURATE.AI platform has been successfully implemented in a variety of disease models ranging from in vitro, to preclinical and clinical trials where CURATE.AI has been validated across indications ranging from immunosuppression to oncology. Additional clinical trial has been approved for using CURATE.AI for drug dosing in the treatment of multiple myeloma. Recently, our team has successfully conducted a proof-of-concept study where CURATE.AI was used to develop N-of-1 learning trajectory profiles.

Furthermore, CURATE.AI identifies drug-dose combinations that optimise safety and efficacy and reside well within clinically acceptable dosing ranges. CURATE.AI is not automated and combination-dosing identification is always performed in consultation between the clinical and CURATE.AI teams to ensure patient safety. The dosing limits and increments will always conform to established clinical guidelines.

8.2. Study Drug Formulations

As per standard of care.

8.3. Study Drug Administration

As per standard of care.

8.4. Specific Restrictions / Requirements

None other than what is already clinically indicated i.e. standard of care.

8.5. Blinding

N/A, no blinding in this study.

8.6. Concomitant therapy

None other than what is already clinically indicated in the standard of care.
All concomitant therapy will be documented accordingly. None are prevented from trial perspective, with recommendations being according to clinical standards-of-care regarding concomitant medication.

8.7. Treatment interruption

In the event of treatment interruption lasting beyond 28 days, there will be a case-review discussion meeting with the principal investigator.

9. SAFETY MEASUREMENTS

9.1. Definitions

Adverse Event
An adverse event is defined as any untoward medical occurrence in a participant administered a pharmaceutical product and which does not necessarily have a causal relationship with the treatment. An adverse event can therefore be any unfavourable and unintended sign (including an abnormal laboratory finding), symptom, or disease temporally associated with the use of a medicinal (investigational) product, whether or not the event is considered causally related to the use of the product. Such an event can result from use of the drug as stipulated in the protocol or labelling, as well as from accidental or intentional overdose, drug abuse, or drug withdrawal. Any worsening of a pre-existing condition or illness is considered an adverse event (with the exception of cancer). Clinical signs and symptoms of disease progression are considered adverse events. Laboratory abnormalities and changes in vital signs are considered to be adverse events only if they result in discontinuation from the study, necessitate therapeutic medical intervention, meet protocol specific criteria and/or if the investigator considers them to be adverse events.

CTCAE term (adverse event description) and grade: The descriptions and grading scales found in the revised NCI Common Terminology Criteria for Adverse Events (CTCAE) version 4.0 will be utilised for adverse event reporting. A copy of the CTCAE version 4.0 can be downloaded from the CTEP web site (http://ctep.cancer.gov/reporting/ctc.html).

• **"Expectedness"**: Adverse events can be "Expected" (see in Sect. 7.1 above) or unexpected.
• **Attribution** of the adverse event:
- Definite – The adverse event *is clearly related* to the study treatment.
- Probable – The adverse event *is likely related* to the study treatment.
- Possible – The adverse event *may be related* to the study treatment.
- Unlikely – The adverse event *is doubtfully related* to the study treatment.
- Unrelated – The adverse event *is clearly NOT related* to the study treatment.

Serious Adverse Event
A serious adverse event or serious adverse drug reaction is any untoward medical occurrence at any dose that meets any of the following criteria, whether related to study drug or not:
Death of Participant An event that results in the death of a participant.
Life-Threatening An event that, in the opinion of the investigator, would have resulted in immediate fatality if medical intervention had not been taken. This does not include an event that would have been fatal if it had occurred in a more severe form.
Hospitalization An event that results in an admission to the hospital for any length of time. This does not include an emergency room visit or admission to an out-patient facility.
Prolongation of Hospitalization An event that occurs while the study participant is hospitalised and prolongs the participant's hospital stay.
Congenital Anomaly An anomaly detected at or after birth, or any anomaly that results in fetal loss.
Persistent or Significant Disability/Incapacity An event that results in a condition that substantially interferes with the activities of daily living of a study participant. Disability is not intended to include experiences of relatively minor medical significance such as headache, nausea, vomiting, diarrhoea, influenza, and accidental trauma.
Important Medical Event Requiring Medical or Surgical Intervention to Prevent Serious Outcome An important medical event that may not be immediately life-threatening or result in death or hospitalization, but based on medical judgment may jeopardise the participant and may require medical or surgical intervention to prevent any of the outcomes listed above (*i.e.,* death of participant, life-threatening, hospitalization, prolongation of hospitalization, congenital anomaly, or persistent or significant disability/incapacity). Examples of such events include allergic bronchospasm requiring intensive treatment in an emergency room or at home, blood dyscrasias or convulsions that do not result in inpatient hospitalization, or the development of drug dependency or drug abuse.
Spontaneous Abortion Miscarriage experienced by study participant.
Elective Abortion Elective abortion performed on study participant.

9.2. Collecting, Recording and Reporting of "Unanticipated Problems Involving Risk to Subjects or Others" – UPIRTSO events to the NHG Domain Specific Review Boards (DSRB)

UPIRTSO events refers to problems, in general, to include any incident, experience, or outcome (including adverse events) that meets ALL of the following criteria:

1. **Unexpected**
   In terms of nature, severity or frequency of the problem as described in the study documentation (eg: Protocol, Consent documents etc).

2. **Related or possibly related to participation in the research**

Possibly related means there is a reasonable possibility that the problem may have been caused by the procedures involved in the research; and

3. **Risk of harm**
Suggests that the research places participant or others at a greater risk of harm (including physical, psychological, economic, or social harm) than was previously known or recognised.

Reporting Timeline for UPIRTSO Events to the NHG DSRB.

1. Urgent Reporting: All problems involving local deaths, whether related or not, should be reported immediately – within 24 hours after first knowledge by the NHG investigator.

2. Expedited Reporting: All other problems must be reported as soon as possible but not later than 7 calendar days after first knowledge by the NHG investigator.

9.3. Collecting, Recording and Reporting of Serious Adverse Events (SAEs) to the Health Science Authority (HSA)

1. For Industry sponsored Trials

All SAEs will be reported to HSA according to the HSA Guidance for Industry "Safety Reporting Requirements for Clinical Drug Trials."

2. For Principal Investigator initiated Trials

All SAEs that are unexpected and related to the study drug must be reported to HSA.

"A serious adverse event or serious adverse drug reaction is any untoward medical occurrence at any dose that:

- Results in death.
- Is life-threatening (immediate risk of death).
- Requires inpatient hospitalization or prolongation of existing hospitalization.
- Results in persistent or significant disability/incapacity.
- Results in congenital anomaly/birth defect.
- Is a Medically important event.

Medical and scientific judgment should be exercised in determining whether an event is an important medical event. An important medical event may not be immediately life threatening and/or result in death or hospitalization. However, if it is determined that the event may jeopardise the participant and/or may require intervention to prevent one of the other adverse event outcomes, the important medical event should be reported as serious."

All SAEs that are unexpected and related to the study drug will be reported. The investigator is responsible for informing HSA no later than 15 calendar days after first knowledge that the case qualifies for expedited reporting. Follow-information will be actively sought and submitted as it becomes available. For fatal or life-threatening cases, HSA will be notified as soon as possible but no

later than 7 calendar days after first knowledge that a case qualifies, followed by a complete report within 8 additional calendar days.

9.4. Safety Monitoring Plan

The principal investigator (PI) is responsible for appropriate medical care of participants during the study. Co-investigators will review all participants before each treatment cycle for monitoring of toxicity. If any participants reports an adverse event, the investigator should follow the participants until the event is either resolved or assessed as stable.

For the prospective study, the medical team will strip the data of any unnecessary personal data information and code it per participants. The code key will be kept in a standalone computer. The coded data will be provided to the data analysis team members the same day as receiving the readout results by the medical team.

The medical team will enlist a staff member to act as a trusted third party and de-identify the data. The aggregated, de-identified data will be provided to the data analysis team members to prevent the re-identification of the participants.

9.5. Complaint Handling

Patients will be advised as per the informed consent form (Appendix 1) that they may contact the Principal Investigator or DSRB secretariat if they have any complaints.

10. DATA ANALYSIS

10.1. Data Quality Assurance

Data integrity will be assured by matching and verifying with the data source. The PI will be subjected to HSA and DSRB audits when needed.

10.2. Data Entry and Storage

A REDCap (Research Electronic Data Capture) database will be established specifically to collect the data for the registry. Each patient recruited into the study will be assigned a unique patient number (UPN), and the patient's biosamples will be labelled using the UPN, with no direct reference to the patient's other identifying information. Information from the source documents will be transcribed onto an electronic database that is password protected in a user designated and password protected computer in the Department. Personnel in the laboratory have no direct access to the clinical history database or other patient information. Information pertaining to the patient that arises from the research will not become part of the patient's medical record. All records will be kept for a minimum period of 6 years following the date of study closure according to ICH GCP guidelines, or longer as applicable per institution guidelines.

11. SAMPLE SIZE AND STATISTICAL METHODS

11.1. Determination of Sample Size

Since this is a pilot clinical trial with no precedent data, we did not perform an upfront formal sample size calculation. We intent to recruit 10 patients, based on the feasibility of recruitment given the rare occurrence of this disease.

11.2. Statistical and Analytical Plans

We will perform and report descriptive analyses of the outcome measures. We will also perform graphical analyses of the temporal variations in response marker level. We will not statistically analyse efficacy and toxicity exploratory outcomes. Additionally, the data collected under this trial may be used to perform analysis towards economic evaluation of personalized dosing with CURATE.AI, such as evaluating the impact of different services and any changes in them due to CURATE.AI, including (but not limited to): medications, monitoring, adverse events and hospitalizations, clinical visits, and diagnostic procedures. This will not require additional data collection.

Upon the end of the trial, explorative analyses will be performed and assessed according to the endpoints.

1.) The response marker data collected (E.g. IgM and/or sFLC) in response to data modulation and at a higher frequency than standard-of-care will undergo descriptive and graphical analysis, among others.

2.) Efficacy and safety measures on dose modulation will be performed via descriptive and graphical analysis, among others.

3.) The suitability of M-proteins quantified from mass spectrometry analysis as an experimental response marker to act as an input in the CURATE.AI analysis will be assessed based on multiple factors, including (but not limited to):

i. Observed dose-dependency;
ii. Changes to mass spectrometry quantified M-proteins within each drug dosing cycle (e.g. half-life and retention);
iii. Specificity and sensitivity of the mass spectrometry quantified M-proteins measurements;
iv. Mass spectrometry quantified M-proteins read-outs range and resolution;
v. Practical aspects of M-proteins measurements via mass spectrometry in multiple myeloma therapy.

12. ETHICAL CONSIDERATIONS

12.1. Informed Consent

No investigator may involve a human being in research unless the investigator has obtained the legally effective informed consent of the patient or the patient's legally authorised representative. An investigator shall seek such consent only under circumstances that provide the prospective patient or the patient's legally authorised representative sufficient opportunity to consider whether or not to participate, and minimise the possibility of coercion or undue influence. The information that is given to the patient or the representative shall be in a language understandable to the patient or representative.

Before implementing any study procedure, informed consent will be documented in the participant case histories and by the use of a written consent form approved by the DSRB and signed and dated by the patient or the patient's legally authorised representative at the time of consent. A copy of the signed informed consent will be given to the patient or patient's legally authorised representative. The original, signed consent will be maintained by the investigator and available for inspection by the regulatory authority at any time. In obtaining and documenting informed consent, the investigator will comply with the SGGCP guidelines and the ethical principles that have their origin in the Declaration of Helsinki.

The patient will be informed about the background and aims of the study. The patient will be told of her right to withdraw from the study at any time without any penalty with regards to the continuation of care at this institution and by the same physicians as she chooses. The patient will be told that tissue and blood samples obtained will be assigned unique patient numbers (UPN) to ensure patient confidentiality.

12.2. IRB review

This protocol and the associated informed consent documents will be sent to review and approval by the NHG DSRB (domain B).

12.3. Confidentiality of Data and Patient Records

Protection and privacy of the personal data of individuals are covered under the Personal Data Protection Act 2012. Patient medical information obtained as part of this study is confidential, and must not be disclosed to third parties, except as noted below. The patient may request in writing that medical information be given to his/her personal physician.

The investigator/institution will permit direct access to source data and document by regulatory authorities. The access may consist of study-related monitoring, audits, DSRB reviews and regulatory authority inspection.

A REDCap (Research Electronic Data Capture) database will be established specifically to collect the data for the registry. Each patient recruited into the study will be assigned a unique patient number (UPN), and the patient's biosamples will be labeled using the UPN, with no direct reference to the patient's other identifying information. Information from the source documents will be transcribed

onto an electronic database that is password protected in a user designated and password protected computer in the Department. Personnel in the laboratory have no direct access to the clinical history database or other patient information. Information pertaining to the patient that arises from the research will not become part of the patient's medical record. All records will be kept for a minimum period of 6 years following the date of study closure according to ICH GCP guidelines, or longer as applicable per institution guidelines.

Information collected includes demographic characteristics, cancer history and pathological information, past and present cancer treatment history of the study participant. The participants' progress may be followed up periodically (approximately every 6 months) through the medical records, and subsequent cancer treatments, progression of cancer, and survival outcome will be updated. Important treatment information that may be collected include: the drug regimens, drug doses, intent of treatment, haematologic and non-haematologic toxicities, and hospitalization episodes that may be related to treatment. Participant may be followed-up till death. Toxicities will be graded using the Common Terminology Criteria for Adverse Events version 4.03 (CTCAE).

Leftover samples are retained for future research for the purpose of:

1. Retrospectively exploring mass-spectrometry quantified M proteins as a suitable marker for CURATE.AI, mass-spectrometry quantified M proteins in comparison to the standard of care markers IgM and/or sFLC.

2. Retaining leftover blood samples for retrospective analysis during or after the trial has ended for the objectives stated in the protocol.

These samples will be kept for not exceeding 30 years after the end of trial and will be de-identified before passing to the study team for analysis.

13. PUBLICATIONS

The research team will submit the study results for publication in peer-reviewed scientific journals. The team's publication policy is aligned with the Consolidated Standards of Reporting Trials (CONSORT) group. No personal health identifiers will be published.

14. RETENTION OF TRIAL DOCUMENTS

Source Documents

Original documents, data, and records (e.g. medical records, raw data collections forms, pharmacy dispensing records, recorded data from automated instruments, laboratory data) that are relevant to the clinical study will be adequately prepared and maintained. These documents are designed to record all observations and other pertinent data for each participant enrolled in this clinical study. Source records will adequately reconstruct all data entered into the case report forms, which will be completed in English.

Archival of Records

The investigators will retain records required to be maintained under this part for a period of 15 years following the completion or discontinuation of the study. The investigators will retain protocols, amendments, IRB approvals, copies of the signed and dates consent forms, medical records, case report forms, drug accountability records, all correspondence, and any other documents pertaining to the conduct of the study.

References:

1. Norton, L. The norton-simon hypothesis revisited. *Cancer Treat. Rep.* **70**, 163 (1986).

2. Peters, W.P.*, et al.* Prospective, randomized comparison of high-dose chemotherapy with stem-cell support versus intermediate-dose chemotherapy after surgery and adjuvant chemotherapy in women with high-risk primary breast cancer: a report of CALGB 9082, SWOG 9114, and NCIC MA-13. *J. Clin. Oncol.* **23**, 2191-2200 (2005).

3. Frei 3rd, E., Elias, A., Wheeler, C., Richardson, P. & Hryniuk, W. The relationship between high-dose treatment and combination chemotherapy: the concept of summation dose intensity. *Clin. Cancer. Res.* **4**, 2027-2037 (1998).

4. Beach, W.A. Managing "Stable" Cancer News. *Soc. Psychol. Q.* **84**, 26-48 (2021).

5. Thummel, K.E. & Lin, Y.S. Sources of interindividual variability. *Methods Mol. Biol.* **1113**, 363-415 (2014).

6. Chakraborty, R., Kapoor, P., Ansell, S.M. & Gertz, M.A. Ibrutinib for the treatment of Waldenström macroglobulinemia. *Expert Rev. Hematol.* **8**, 569-579 (2015).

7. Gribben, J.G.*, et al.* Optimising outcomes for patients with chronic lymphocytic leukaemia on ibrutinib therapy: European recommendations for clinical practice. *Br. J. Haematol.* **180**, 666-679 (2018).

8. Chen, L.S.*, et al.* A pilot study of lower doses of ibrutinib in patients with chronic lymphocytic leukemia. *Blood.* **132**, 2249-2259 (2018).

9. Bose, P., Gandhi, V.V. & Keating, M.J. Pharmacokinetic and pharmacodynamic evaluation of ibrutinib for the treatment of chronic lymphocytic leukemia: rationale for lower doses. *Expert Opin. Drug Metab. Toxicol.* **12**, 1381-1392 (2016).

10. Finnes, H.D.*, et al.* Pharmacovigilance during ibrutinib therapy for chronic lymphocytic leukemia (CLL)/small lymphocytic lymphoma (SLL) in routine clinical practice. *Leuk. Lymphoma* **58**, 1376-1383 (2017).

11. Byrd, J.C.*, et al.* Acalabrutinib Versus Ibrutinib in Previously Treated Chronic Lymphocytic Leukemia: Results of the First Randomized Phase III Trial. *J. Clin. Oncol.* **39**, 3441-3452 (2021).

12. Buske, C., Jurczak, W., Salem, J.-E. & Dimopoulos, M.A. Managing Waldenström's macroglobulinemia with BTK inhibitors. *Leukemia* **37**, 35-46 (2023).

Date:

Re: PRECISE clinical trial - CURATE.AI dose recommendation

Patient's Code:
Regimen:
Modulated Drug:

Cycle:
Cycle tentative start day:
Recommendation No.:
Recommendation Intent:

CURATE.AI Team recommends the total of mg of Ibrutinib (equivalent of days of 420 mg daily and days of 280 mg daily) over the duration of the 4-week cycles.

The recommendation is approved for administration by the clinical investigator:

Name & Signature

Date
